# *Pseudomonas aeruginosa* LasB Subverts Alveolar Macrophage Activity by Interfering With Bacterial Killing Through Downregulation of Innate Immune Defense, Reactive Oxygen Species Generation, and Complement Activation

**DOI:** 10.3389/fimmu.2018.01675

**Published:** 2018-07-23

**Authors:** Fabien Bastaert, Saadé Kheir, Vinciane Saint-Criq, Bérengère Villeret, Pham My-Chan Dang, Jamel El-Benna, Jean-Claude Sirard, Romé Voulhoux, Jean-Michel Sallenave

**Affiliations:** ^1^INSERM, UMR1152, Paris, France; ^2^Laboratoire d’Excellence Inflamex, Département Hospitalo-Universtaire FIRE (Fibrosis, Inflammation and Remodeling), University Paris Diderot, Sorbonne Paris Cité, Paris, France; ^3^INSERM UMR1149, ERL 8252 CNRS, Centre de Recherche sur l’Inflammation, Paris, France; ^4^Université Paris Diderot, Sorbonne Paris Cité, Laboratoire d’Excellence Inflamex, Faculté de Médecine, Site Xavier Bichat, Paris, France; ^5^Centre d’Infection et d’Immunité de Lille, Institut Pasteur de Lille, INSERM, U1019, Lille, CNRS, UMR 8204, Université de Lille, Lille, France; ^6^CNRS & Aix-Marseille Université, Laboratoire d’Ingénierie des Systèmes Macromoléculaires (UMR7255), Institut de Microbiologie de la Méditerranée (IMM), Marseille, France

**Keywords:** alveolar macrophage, *Pseudomonas aeruginosa*, LasB, lung, infection, inflammation, cystic fibrosis, nosocomial infections

## Abstract

*Pseudomonas aeruginosa* (*P.a*) is a pathogen causing significant morbidity and mortality, in particular, in hospital patients undergoing ventilation and in patients with cystic fibrosis. Among the virulence factors secreted or injected into host cells, the physiopathological relevance of type II secretions system (T2SS) is less studied. Although there is extensive literature on the destructive role of LasB *in vitro* on secreted innate immune components and on some stromal cell receptors, studies on its direct action on myeloid cells are scant. Using a variety of methods, including the use of bacterial mutants, gene-targeted mice, and proteomics technology, we show here, using non-opsonic conditions (thus mimicking resting and naïve conditions in the alveolar space), that LasB, an important component of the *P.a* T2SS is highly virulent *in vivo*, and can subvert alveolar macrophage (AM) activity and bacterial killing, *in vitro* and *in vivo* by downregulating important secreted innate immune molecules (complement factors, cytokines, etc.) and receptors (IFNAR, Csf1r, etc.). In particular, we show that LasB downregulates the production of C3 and factor B complement molecules, as well as the activation of reactive oxygen species production by AM. In addition, we showed that purified LasB impaired significantly the ability of AM to clear an unrelated bacterium, namely *Streptococcus pneumoniae*. These data provide a new mechanism of action for LasB, potentially partly explaining the early onset of *P.a*, alone, or with other bacteria, within the alveolar lumen in susceptible individuals, such as ventilated, chronic obstructive pulmonary disease and cystic fibrosis patients.

## Introduction

*Pseudomonas aeruginosa* (*P.a*) is an important bacterial lung pathogen, which causes significant morbidity and mortality, in particular, in hospital patients undergoing ventilation, and in patients with cystic fibrosis (CF) (1, 2). *P.a* can express a plethora of virulence factors, through different secretion systems, abbreviated T1-T6 SS (3).

The respective role of these in *P.a* pathogenesis has not been extensively investigated, particularly *in vivo*. Recently, Jyot et al. (4) demonstrated, using *P.a* deleted for the T2 or T3 SS (PAK strains) that PAK strain possessing the T3SS were highly virulent, but they also demonstrated the T2SS to have an unidentified virulent effect on mice host defenses, which manifested itself by a reduced lung bacterial clearance. Because the T2SS is composed of many proteins, it remained important to attempt the identification of factors potentially responsible for this effect. Relatedly, we have shown recently (5) that the T2SS metalloprotease LasB, an important virulence factor present in CF secretions (6–9) and chronic obstructive pulmonary disease (COPD) exacerbations (10), is by far the most abundant protein in WT-PAO1 *P.a* secretome, and hence hypothesized here that LasB may be one of the factors implicated in that subversion.

Indeed, it had been shown previously by us and others that LasB was able to degrade cytokines, eukaryotic cell surface receptors, and to participate in bacterial escape from the host immune system, leading to host colonization, and tissue destruction (5, 11–14). Surprisingly, relatively little was known about the direct effect of LasB in the lung (15–17).

We show here that following intra-nasal administration in mice, WT-PAO1 (expressing LasB) is highly virulent when compared to ΔLasB-PAO1, and that there is a significant difference in lung bacterial recovery between the two strains, at an early time point (2 h) when inflammatory cell influx is still absent. We, therefore, focused the rest of our study on the alveolar macrophage (AM) and identify here for the first time AM as an important target for LasB subversion of innate immune defenses in the lung. After ruling out an effect of LasB on TLR-5, a receptor that we demonstrated previously to be important for the recognition of *P.a* by AM (18), we demonstrate, through proteomics and immune analysis, that LasB downregulates, in particular, the production of complement C3 and factor B components, as well as the activation of reactive oxygen species (ROS) production by AM. In addition, we show that purified LasB impaired significantly the ability of AM to clear an unrelated bacterium, namely *Streptococcus pneumoniae*. These data provide a new mechanism of action for LasB, potentially partly explaining the early onset of *P.a*, alone, or with other bacteria, within the alveolar lumen in susceptible individuals (such as ventilated, COPD and cystic fibrosis patients), where a deficit in phagocytic/killing activity in alveolar macrophages (AMs) has been implicated (2).

## Materials and Methods

### Chemicals and Enzymes

Phosphoramidon (PA), a specific metalloprotease inhibitor was obtained from Sigma. Neutrophil elastase (NE) was obtained from Elastin products.

### Bacterial Strains and Products

PAO1 was obtained from the ATCC (15692). WT-PAO1 and LasB-deleted strains were transformed with the plasmid pML27 (19) coding for LasB (referred to as pLasB thereafter), or with the plasmid control pML27, referred to as p0 thereafter. PAO1 strains were kept in freezing medium (50% LB, 50% glycerol) until use and *S. pneumoniae* (clinical isolate E1586) was stored in −80°C in freezing medium (50% Todd Hewitt Yeast THYB/50% Glycerol).

Before infection experiments, PAO1 and *S. pneumoniae* strains were grown overnight in LB and THYB broths, respectively. After dilution and growth (until reach of the exponential growth phase), OD was measured at 600 nm. Bacteria were then centrifuged (3,400 *g* for 15 min) and pellets resuspended at the desired multiplicity of infection (moi) or colony forming units (cfu) in PBS.

In parallel, bacterial supernatants (secretomes = SEC) were filter-sterilized using a 0.2 µm pore filter, aliquoted, and stored at −80°C until further use.

Purified LasB (referred to as LasB thereafter) was a kind gift of Pr. G. Döring (University of Tubingen, Germany). WT *Salmonella typhi murium* flagellin was obtained in house (JCS’s laboratory) and LPS was obtained from Enzo Life Science.

### *In Vivo* and *Ex Vivo* Macrophage Experiments

After anesthesia (using intraperitoneal injection of a mixture of ketamine–xylazine), 6- to 10-week-old male C57BL/6JRj mice were infected intra-nasally (i.n) with 10^9^ cfu of *P.a* bacterial strains (see above) and survival was followed over time. During the whole course of *in vivo* experiments, animals were maintained under 12 h light/dark cycles, with free access to food and water. All efforts were made to minimize suffering and all experimental procedures were performed in a Biosafety Level 3 facility, following the European Union guidelines for the handling of laboratory animals.

Alternatively, in independent experiments, sub-lethal doses (5 × 10^6^ cfu) of *P.a* strains were used, and at various time points (2, 4, 20 h), mice were euthanized with pentobarbital, tracheae were cannulated, and a bronchoalveolar lavage (BAL) (2 mL of total volume) was performed. BAL fluid (BALF) supernatant was kept at −80°C until further use, e.g., for cytokines/chemokines measurement, using DuoSet enzyme-linked immunosorbent assay kits (R&D Systems) or for enzymatic analysis (LasB, NE). Simultaneously, the BAL cell pellet was resuspended in 400 µL of PBS for cell type analysis, using cytospin centrifugation and Diff-Quik staining (Medion Diagnostics). In parallel, lungs were recovered in 1 mL of PBS and homogeneized with a FastPrep-24 (MP Biomedicals) during two cycles (speed 6, 45 s). Homogenates were then used for bacterial counts (on agar plates) or for cytokine/chemokines/enzymatic measurements.

In a different set of experiments, AMs were recovered for *ex vivo* experiments through lavaging the lungs in PBS/EDTA 0.5 mM (see above), and cultured in RPMI medium containing 10% FCS, antibiotics and antimycotics (1,000 U/mL penicillin, 100 µg/mL streptomycin and 0.25 µg/mL Amphotericin B, Themo Fischer Scientific) before being placed in serum-free RPMI medium, and stimulated or infected during 4 h.

### *In Vitro* Macrophage Experiments

#### Macrophage Cell Lines

– THP-1 cells (TIB-202™; ATCC) were cultured in complete RPMI medium (supplemented with 2 mM l-glutamine, 1% antibiotic, and 10% inactivated FBS). Prior to infection or challenge, THP-1 cells were differentiated into macrophages with a 3 h phorbol 12-myristate 13-acetate treatment (PMA 0.5 µM, Sigma), washed, and plated overnight. Differentiated THP-1 was placed into serum- and antibiotic-free RPMI for 4 h before stimulation/infection.
– The MPI alveolar cell line [gift from Dr. G. Fejer (20)] was cultured as above at 37°C in a 5% CO_2_ in RPMI medium containing 10% FCS and 30 ng/mL of mGM-CSF. As above, 4 h prior to infection/stimulation, MPI cells were placed in serum-free RPMI medium.

#### AMs and MPIs Killing of *P.a*

10^5^ macrophages (THP-1, MPI, or AMs) were infected with *P.a* (moi = 1) during 4 h in 96-well plates and cultured in RPMI medium (200 μL/well), as indicated above. In parallel, “control wells (C well)” (with no macrophages) were set-up to determine the baseline bacterial growth during 4 h. At the end of the experiment, supernatants (S) (0.2 mL diluted with 1.4 mL of PBS) were added to the cell layer (L), lysed with 400 µL of PBS containing 0.1% of Triton X-100 (i.e., L + S, Triton 0.02%, a concentration, which *per se* does not lyse bacteria, not shown). Remaining bacteria were then counted in L + S as above on LB-agar plates. Bacterial killing was calculated by counting the remaining CFU in L + S at 4 h and normalized to the CFU obtained when the inoculum was seeded at T0 in the absence of macrophages. A value of 1 was given to the maximal bacterial clearance obtained with the WT strain of PAO1, and other treatments were then expressed relative to 1.

### MPIs Killing of *S. pneumoniae*

MPI cells were plated as above and incubated for 2 h with either medium alone (serum-free RPMI) or medium supplemented with 50 nM recombinant LasB. After washing, cells were infected in serum-free RPMI with *S. pneumoniae* (MOI 1) for 4 h. In parallel, the bacterial inoculum was seeded at T0 in the absence of macrophages as a control of bacterial growth, as in Section “AMs and MPIs Killing of *P.a*.”

For bacterial count and bacterial clearance measurements, supernatants were collected and cells were lysed in PBS-Triton as above. 100 µL of each dilution were spread on THYB-Agar plates and left 48 h at 37°C, 5% CO_2_ for CFU count. As above, a value of 1 was given to the maximal bacterial clearance obtained with the *S. pneumoniae*, and other treatments were then expressed relative to 1.

### Bone Marrow-Derived Macrophages (BMDM) Preparation

Bone marrow was extracted from mice femurs after PBS injection. Cells were washed with PBS, centrifuged at 1,400 rpm for 7 min (4°C). Pellet cells were then resuspended with lysis buffer (Gibco) during 2 min at room temperature to lyse red blood cells. After another wash, cells were seeded in complete DMEM medium (10% FCS, Pen/Strep and gentamycine) containing 30 ng/mL of mouse M-CSF (Preprotech). After two successive medium replacement (at D3 and D6), macrophages were resuspended at D9 in cold PBS, and plated in 96-well plates for PAO1 infection (same conditions as for other macrophages, see above).

### ROS Production

Reactive oxygen species production was measured by the luminol-enhanced chemiluminescence method: briefly, 7 × 10^5^ MPI cells were resuspended in 500 mL of HBSS containing 10 µmol/L luminol (Sigma) preheated to 37°C in the thermostated chamber of the luminometer (Biolumat LB937; Berthold). Opsonized zymosan (Sigma), secretomes, or diphenyleneiodonium (DPI) were added at the indicated concentrations and chemiluminescence was recorded for 90 min.

### Cell Cytotoxicity Measurement

Cell cytotoxicity was measured through the quantification of lactate dehydrogenase (LDH) ratio in cell supernatants versus cell lysates (PBS/Triton X-100 (0.8%)). This was performed in 96-well plates (Costar) and absorbance was measured at 490 nm (kit *CytoTox96^®^Non Radioactive Cytotoxicity Assay kit* (Promega) in a *Multiskan Ascent* (Thermo Scientific) plate reader). Data were treated using the *Ascent Software (Version 2.6)*, with % cytotoxicity = {OD of supernatant/(OD of supernatant + OD lysate)} × 100.

### Enzymatic Activities

All enzymatic activities were performed in 384 black wells plates in a final volume of 30 µL. All data were analyzed with the software *SkanIt Software (Version 2.2.184)*.

#### LasB Assay

LasB bioactivity was determined using a fluorogenic substrate specific for metalloproteases, TACE substrate II [5-FAM-Ser-Pro-Leu-Ala-Gln-Ala-ValArg-Ser-Ser-Ser-Arg-Lys(5-TAMRA)-NH2; Enzo Life Sciences: excitation and emission wavelength being 485 and 535 nm, respectively]. Purified LasB and samples (diluted in Tris 25 nM; ZnCl_2_ 2.5 µM; Brij-35 0.005%; pH 9.0 buffer) were incubated at room temperature in the presence of substrate (10 µM) and fluorescence was read over a 3 h period (one measure taken every 10 min) with a *Varioskan*™ *Flash Multimode Reader* (Thermo Scientific).

#### NE Assay

Briefly, purified NE and BAL samples (diluted in Tris 50 mM; NaCl 0.5 M; Triton X-100; 0.1%; pH 8.0) were incubated at room temperature as above (LasB assay) in the presence of 0.1 mg/mL NE substrate (Methoxysuccinyl-Ala-Ala-Pro-Val-7-amido-4-methylcoumarin, Sigma, excitation and emission wavelength being 460 and 370 nm, respectively), and fluorescence was read over a 3 h period, as above (21).

### Enzyme-Linked Immunosorbent Assays (ELISAs)

The secretion of cytokines and complement factors (C3, factor B) from MPI cells or from *in vivo* experiments was quantified by ELISA, according to the manufacturers’ instructions: R&D Systems (for IL-1β and TNF-α), Abcam plc (for C3), or MyBiosource (for factor B).

### Proteomics Mass Spectrometry Analysis

For proteomics analysis, MPI cells were cultured in 60 mm culture dishes (5.5 × 10^6^ cells) in 5 mL of serum-containing RPMI. When subconfluent, cells were either treated with serum-free RPMI (control, denoted “MPI” in the Proteomics analysis), or infected with either WTp0-PAO1 or ΔLasBp0-PAO1 in the same serum-free medium during 4 h (moi = 1). Supernatants were then collected, and concentrated to 1 mL with Ultracell YM-3 (cut-off of 3 kDa) centrifugal filters (Millipore). Samples were then provided to the Mass Spectrometry Laboratory [Institut Jacques Monod (UMR 7592, Univ Paris Diderot, CNRS, Sorbonne Paris Cité] for LC-MS/MS acquisition. Protein extracts (30 µg) of the different conditions were precipitated with acetone at −20°C during 3 h and incubated with 20 µL of 25 mM NH_4_HCO_3_ containing sequencing-grade trypsin (12.5 µg/mL; Promega) overnight at 37°C. Peptides were desalted using ZipTip μ-C18 Pipette Tips (Millipore) and analyzed in biological triplicates by a Q-Exactive Plus coupled to a Nano-LC Proxeon 1000 equipped with an easy spray ion source (all from Thermo Scientific). Peptides were separated by chromatography with the following parameters: Acclaim PepMap100 C18 pre-column (2 cm, 75 µm i.d., 3 µm, 100 Å), Pepmap-RSLC Proxeon C18 column (50 cm, 75 µm i.d., 2 µm, 100 Å), 300 nL/min flow rate, gradient from 95% solvent A (water, 0.1% formic acid) to 35% solvent B (100% acetonitrile, 0.1% formic acid) over a period of 98 min, followed by a column regeneration for 23 min, giving a total run time of 2 h. Peptides were analyzed in the Orbitrap cell, in full ion scan mode, at a resolution of 70,000 (at *m/z* 200), with a mass range of *m/z* 375–1,500 and an AGC target of 3 × 10^6^. Fragments were obtained by high collision-induced dissociation activation with a collisional energy of 30%, and a quadrupole isolation window of 1.4 Da. MS/MS data were acquired in the Orbitrap cell in a Top20 mode, at a resolution of 17,500, with an AGC target of 2 × 10^5^, with a dynamic exclusion of 30 s. MS/MS of most intense precursor were first acquired. The maximum ion accumulation times were set to 50 ms for MS acquisition and 45 ms for MS/MS acquisition. Label-free relative quantification was performed in between subject analysis using Progenesis-Qi software 3.0 (Nonlinear Dynamics Ltd.). For the identification step, all MS and MS/MS data were processed with the Proteome Discoverer software (Thermo Scientific, version 2.1) coupled to the Mascot search engine (Matrix Science, version 2.5.1). The mass tolerance was set to 7 ppm for precursor ions and 0.02 Da for fragments. The maximum number of missed cleavages was limited to two for the trypsin protease. The following variable modifications were allowed: oxidation (Met), phosphorylation (Ser, Thr), acetylation (Protein N-term). The NCBInr database (2016) with the *Mus musculus* and the *P.a* taxonomies were used for the MS/MS identification step. Peptide identifications were validated using a 1% False Discovery Rate (FDR) threshold calculated with the Percolator algorithm. Protein abundance variations were measured according to the Hi-3 label-free quantification method and validated if their calculated ANOVA *p*-values were under 0.05.

Fold increase expression (cut-off ≥ 2, *p* ≤ 0.05) between individual conditions were reported in Excel sheets (see Supplementary Material). When appropriate, Venn diagrams generated from “Proteome Discoverer” or from “Venny”^1^ were given and String^2^ or Reactome^3^ analysis was performed.

### Mice Experiments

Six- to ten-week-old C57BL/6JRj WT mice were obtained from Janvier (Le Genest-Saint-Isle, France). Tlr4−/− and Tlr5−/− on C57BL/6JRj background were bred in house, in a specific pathogen-free facility (#A59-350009, Institut Pasteur de Lille) and experiments complied with national regulations and ethical guidelines.

### Statistics

Statistical analysis of the differences observed between groups (*in vivo*) and experimental conditions (*ex vivo* and *in vitro*) was performed using either a non parametric test of variance (Kruskal–Wallis and Dunn’s posttest) or using a parametric test (one-way ANOVA followed by the appropriate “multicomparison” posttests). Data were analyzed using the Prism^®^ (*Graphpad Prism Version 6.03*) software. In addition, survival curves were analyzed with the Log-rank (Chi^2^) test. Differences were considered statistically significant when *p* was <0.05 and were labeled as follows: *p < 0.05; **p < 0.01; ***p < 0.001; ****p < 0.0001; NS, non significant.

## Results

### C57/Bl6 Mice Inoculated With ΔLasB PAO1 Survive Longer Than Mice Treated With WT PAO1 and With LasB-Complemented Strains

**Figure 1:**
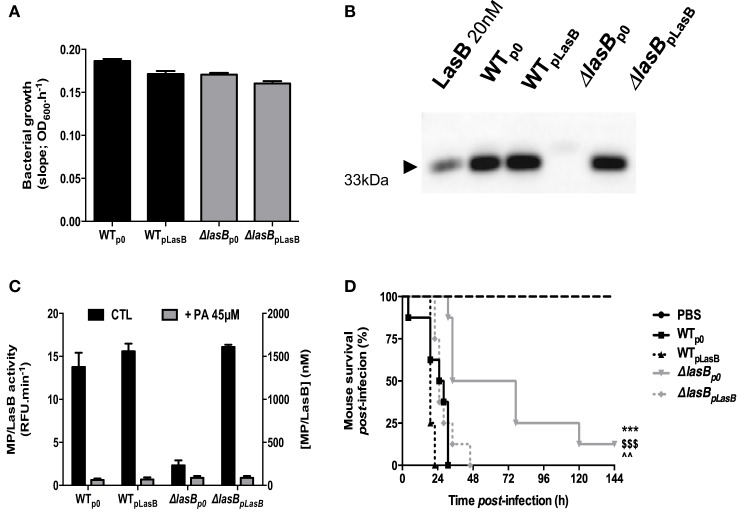
Characterisation of bacterial strains and mice survival. **(A)** The growth of WT−, WT + pLasB−, ΔLasB−, ΔLasB + pLasB-PAO1 was evaluated by measuring the absorbance at 600 nm of overnight bacterial culture supernatants. Results are expressed as means ± SD. **(B)** The presence of LasB in these cultures was confirmed by Western Blot analysis with anti-LasB immun-serum (see Materials and Methods). **(C)** The enzymatic activity of LasB in these cultures was measured using TACE substrate II, a fluorogenic substrate (dark bars), and the specificity of this activity was verified using phosphoramidon (PA), a metalloprotease inhibitor (gray bars, see Materials and Methods). Results are expressed as means ± SD. **(D)** C57/Bl6 mice were infected intra-nasally (i.n) with PBS (*n* = 4) or 10^9^ cfu of *Pseudomonas aeruginosa* bacterial strains (see above, *n* = 8 for each bacterial strain) and mouse survival was followed over time. ***p < 0.001, compared to WT-PAO1; ^$$$^*p* < 0.001, compared to WTplasB; ^^^^*p* < 0.01, compared to ΔLasBpLasB.

All strains: WTp0, ΔLasBp0 (respectively, WT-PAO1 and ΔLasB complemented with the plasmid “null”), WTpLasB, and ΔLasBpLasB-PAO1 (respectively, WT-PAO1 and ΔLasB-PAO1 complemented with the plasmid “pLasB”) grew equally well in LB medium (Figure 1A) and secreted high levels of active LasB, except for ΔLasB, as expected (Figures 1B,C). Notably, PA, a specific metalloprotease inhibitor, inhibited that activity (Figure 1C, gray bars).

In addition, Figure 1D shows that all strains expressing LasB were significantly more virulent than ΔLasBp0-PAO1 and killed mice quicker.

### WT PAO1 Is Cleared Less Efficiently Than ΔLasB PAO1 *In Vivo*, While Inflammatory Cell Recruitment Remains Unchanged

**Figure 2:**
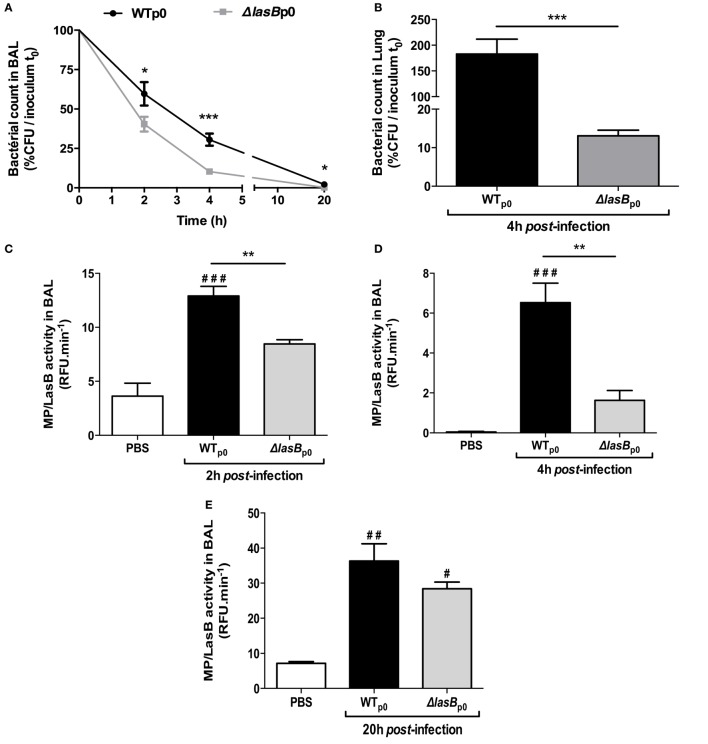
*In vivo* bacterial clearance and enzymatic measurements. C57/Bl6 mice (7 ≤ *n* ≤ 17) were infected i.n with WT-PAO1 or ΔLasB-PAO1 (5 × 10^6^ cfu), and bacterial growth **(A,B)**, metalloprotease activity **(C–E)** were measured in lung extracts and in BAL fluid, as explained in Section “Materials and Methods.” **(A)** Results are expressed as means ± SEM, and statistical significance assessed by Mann–Whitney test. Statistical significance between WT- and ΔLasB at each time point: *p < 0.05; ***p < 0.001; **(B)** results are expressed as means ± SEM, and statistical significance assessed by Mann–Whitney test: ***p < 0.001; **(C)** results are expressed as means ± SEM, and statistical significance assessed by a Kruskal–Wallis test, followed by Dunn’s post-analysis. **p < 0.01 compared to WT-PAO1; ^###^*p* < 0.001, compared to PBS; **(D)** same representation and statistical analysis as in **(C)** **p < 0.01, compared to WT-PAO1; ^###^*p* < 0.001, compared to PBS; **(E)** same representation and statistical analysis as in **(C)** ^#^ and ^##^: *p* < 0.05 and 0.01, respectively, compared to PBS.

We next infected mice *i.n* with WTp0- and ΔLasBp0-PAO1 strains with a sub-lethal dose (5 × 10^6^ cfu) and showed that WTp0- bacterial load in BALF was higher than that of ΔLasBp0-PAO1 at all time points (Figure 2A) and the difference was the highest at 4 h in the lungs of these mice (Figure 2B). Mirroring this, *in vivo* total metalloprotease activity (of which LasB is probably a significant component) was consistently lower in the BAL of ΔLasB PAO1-treated mice over the time course (Figures 2C–E).

**Figure 3:**
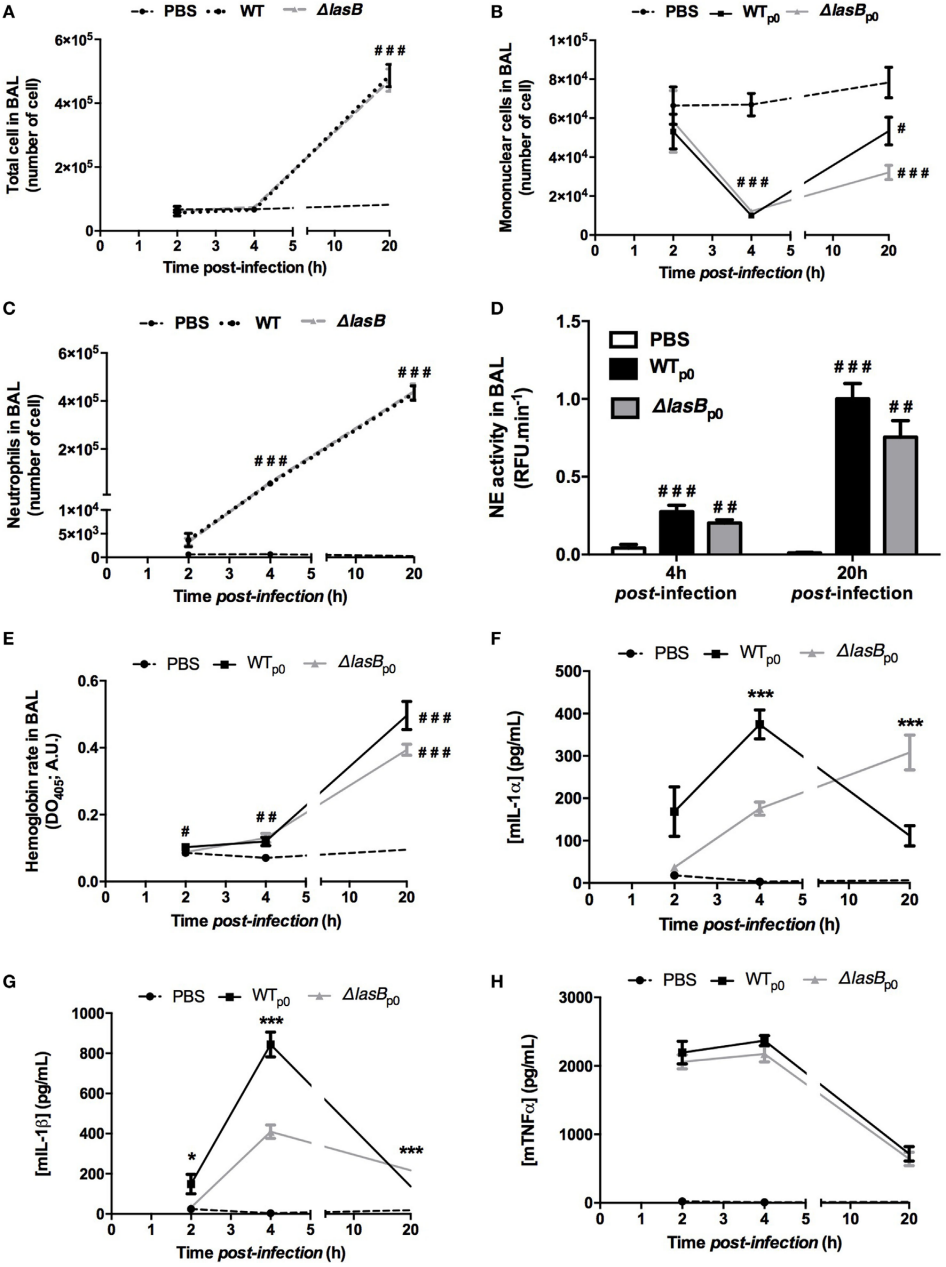
Analysis of inflammatory markers following *in vivo Pseudomonas aeruginosa* infection of C57/Bl6 mice. **(A)** BAL fluid (BALF) samples collected from the experiments depicted in Figure 2 were analyzed, at various time points, for total cell numbers, macrophage/mononuclear and neutrophil numbers **(A–C)**. Also, neutrophil elastase (NE) activity, as an index of neutrophil activation, was measured, using a specific fluorogenic substrate **(D)**. Hemoglobin release, as an index of tissue injury (OD at 413 nm), was also measured **(E)**. Additionally, BALF IL-1-α, IL-1β, and TNF-α levels were measured by enzyme-linked immunosorbent assays [**(F–H)**, respectively]. For all experiments, results are presented as mean ± SEM and ANOVA, followed by Bonferonni tests analysis: Statistical significance: **(A)** ^###^*p* < 0.001 compared to PBS; **(B)** ^#^, ^##^, ^###^: *p* < 0.05, <001, <0.001, respectively, compared to PBS; **(C)** ^###^*p* << 0.001, compared to PBS; **(D)** ^##^, ^###^: *p* < 001 and <0.001, respectively, compared to PBS; **(E)** ^#^, ^##^, ^###^: *p* < 0.05, <001, <0.001, respectively, compared to PBS; **(F,H)** *p < 0.05, ***p < 0.001 compared to PBS.

At the cellular level, PBS vehicle did not, over time, modulate total BAL cell numbers, including AMs content, nor that of neutrophils (Figures 3A–C). However, both PAO1 strains decreased AM numbers, 4 and 20 h post-infection (p.i.) whereas AM numbers were unchanged 2 h p.i. (Figure 3B). Both strains also induced significant neutrophilia, as early as 4 h, up to 20 h (Figure 3C; Figure S1 in Supplementary Material). These neutrophils were activated, as demonstrated by the measurement of significant NE activity in BALF (Figure 3D), but independently of the presence or not of LasB. Although not statistically significant, WT-PAO1 showed a trend for higher virulence (increase in BALF hemoglobin levels at 20 h, Figure 3E). IL-1α, IL-1β levels were higher at early time points (2 and 4 h) in WT-PAO1-treated mice, but this was reversed at 20 h (Figures 3F,G). TNF-a levels were increased by PAO1 infection, but independently of the presence of LasB (Figure 3H).

### Macrophage Clearance of WT PAO1 Is Less Efficient Than That of ΔLasB PAO1

Because the difference in the clearance of PAO1 strains occurred *in vivo* as early as 2 h p.i. (Figure 2A), when the only quantitatively important phagocytic cell is the alveolar macrophage, we focused the rest of our study on that cell type. Indeed, in all the macrophage cell types studied either *ex vivo* (C57Bl/6JRj AMs obtained from BAL and BMDMs obtained from bone marrow) or *in vitro* (MHS, MPI, THP-1 cell lines), we showed after pooling AM supernatants and AM lysates that WTp0 (given a standard clearance value of 1) and ΔLasBpLasB were less cleared than the ΔLasBp0 strain 4 h p.i. (Figures S2A–C in Supplementary Material). We checked that this was not due to differences in cell cytotoxicity (LDH activity, Figure S2D in Supplementary Material), nor to differences in AM phagocytosis (not shown).

**Figure 4:**
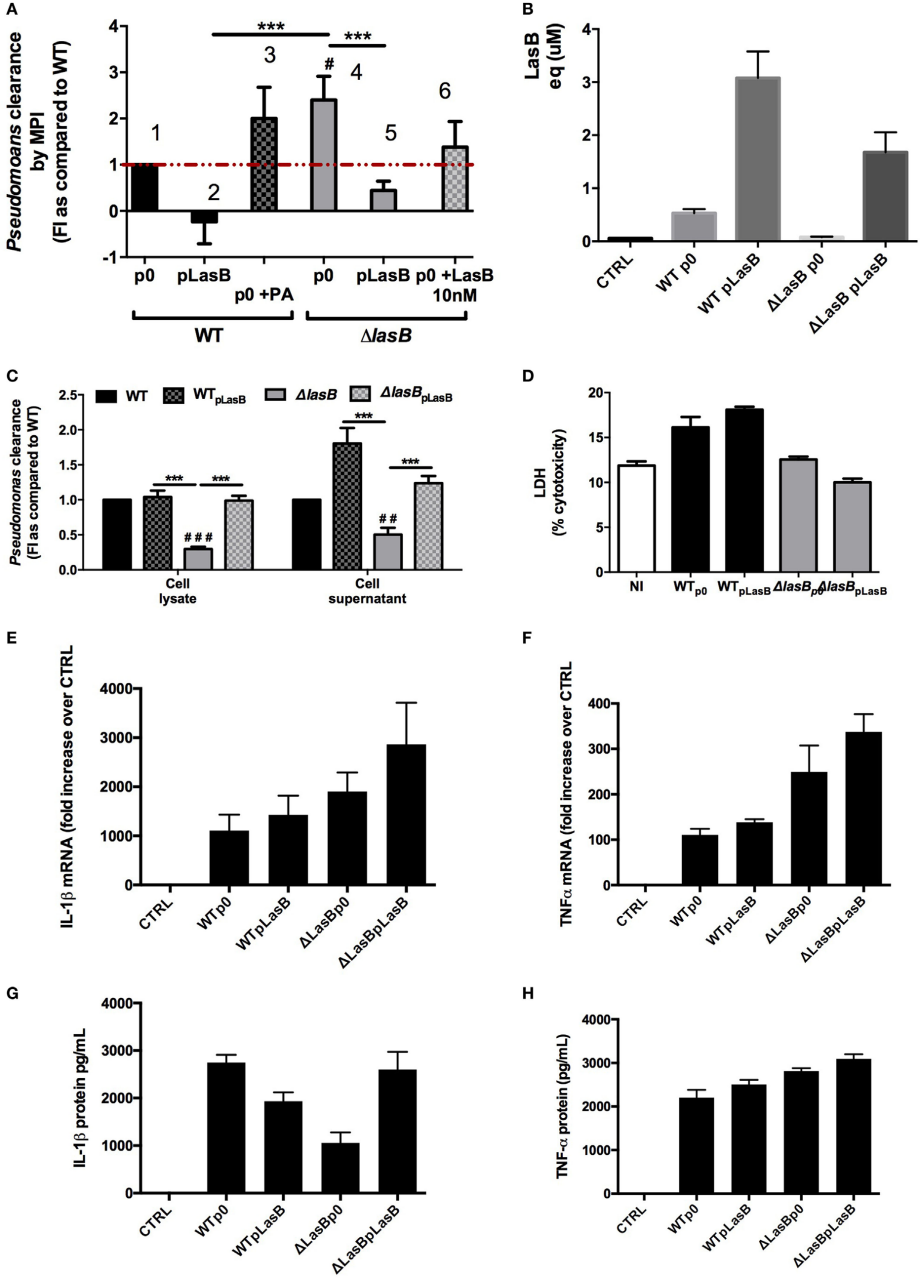
Analysis of bacterial clearance by mouse primary alveolar macrophages and macrophage cell lines. **(A)** MPI cells were plated (10^5^ cells in 96-well plates) and infected (*n* = 6) in serum-free RPMI medium during 4 h (moi = 1) with either WT- + p0/pLasB- (histograms 1–2), ΔLasB- + p0/pLasB- (histograms 4–5), WT-p0 + phosphoramidon- (PA 8.5 µM, histogram 3), or with ΔLasB + p0-PAO1 in the presence of purified LasB protein (10 nM, histogram 6). Then, supernatants and cell lysates (S + L) were pooled and CFU of remaining PAO1 were counted on LB-agar plates. This was compared to the CFU obtained when the inoculum was seeded at T0 in the absence of macrophages. For bacterial clearance, a value of 1 was given to macrophages infected with WT-PAO1, and other treatments were then expressed relative to 1 (see Materials and Methods). Results are expressed as mean ± SEM and statistical analysis performed with Kruskal–Wallis test, followed by Dunn’s post-analysis: ^#^*p* < 0.05, compared to MPI infected by WT + p0-PAO1 alone and ***p < 0.001, compared to MPI infected by ΔLasB + p0-PAO1 alone. **(B)** An aliquot of MPI supernatants **(A)** was measured for metalloprotease activity (see Materials and Methods). The activity was reported as “LasB equivalent micromoles,” comparing it with the activity measured with a purified LasB standard curve. Results are expressed as means ± SEM, and analyzed with one-way ANOVA, followed by Tukey’s post-analysis. CTRL v WTp0: *p* < 0.05; CTRL v WTpLasB: *p* < 0.05; WTp0 v WTpLasB: *p* < 0.05; WTp0 v ΔLasBp0: *p* < 0.05; WTpLasB v ΔLasBp0: *p* < 0.05; WTpLasB v ΔLasBpLasB: *p* < 0.05. **(C)** In an independent experiment (*n* = 6), supernatants and lysates, as explained in **(A)** were assessed separately for bacterial counts. Results are expressed as fold increase over WT + p0-PAO1. Results are expressed as mean ± SEM and statistical analysis performed with a two-way ANOVA test, followed by Sidak’s analysis: Statistical significance: ^##^*p* < 0.01, ^###^*p* < 0.001, compared to WT + p0; ***p < 0.001. **(D)** Cell cytotoxicity measurement of samples generated in **(A)** was performed by measuring the release of lactate dehydrogenase in the supernatant. CTRL = NI = non-infected cells. Results are expressed as mean ± SEM and statistical analysis (no significance) performed with Kruskal–Wallis test, followed by Dunn’s post-analysis. **(E–H)** In an independent experiment (*n* = 3), supernatants and lysates from **(A)** were used for RNA preparation and enzyme-linked immunosorbent assays cytokine assessment; **(E,F)** expression of IL-1β and TNF-α mRNA, respectively (fold increase over CTRL = non-infected cells); **(G,H)** expression of IL-1β and TNF-α protein (pg/ml), respectively. Results are expressed as means ± SEM, and analyzed with one-way ANOVA, followed by Tukey’s post-analysis. **(E)** CTRL v ΔLasBpLasB: *p* < 0.05; **(F)** CTRL v all groups: *p* < 0.05; WTp0 v ΔLasBp0: *p* < 0.001; WTp0 v ΔLasBpLasB: *p* < 0.001; WTpLasB v ΔLasBpLasB: *p* < 0.01; **(G)** CTRL v all groups: *p* < 0.05; WTp0 v ΔLasBp0: *p* < 0.001; ΔLasBp0 v ΔLasBpLasB: *p* < 0.001; **(H)** CTRL v all groups: *p* < 0.05.

To further dissect the mechanisms involved, we used MPI cells [a macrophage cell line with an alveolar phenotype (20)] for the rest of our study, because the differences between WTp0- and ΔLasBp0-PAO1 were most marked in that cell type (Figure S2B in Supplementary Material). MPI cells were infected with all four bacterial strains, i.e., WTp0-, WTpLasB-, ΔLasBp0, and ΔLasBpLasB-PAO1 (Figure 4A), and all strains genetically able to produce LasB secreted recordable metalloprotease activity in the short time course of the experiment (4 h), albeit with different efficiencies, with the “strongest” being the WT-pLasB strain (see legend of Figure 4B for statistical significance).

We confirmed that WTp0- (Figure 4A, histogram 1) was less cleared than ΔLasBp0-PAO1 (histogram 4) and that ΔLasBpLasB (histogram 5) behaved similarly as WTp0- (histogram 1) and WTpLasB- (histogram 2). Furthermore, we showed that LasB was acting through its enzymatic activity: first, PA, an inhibitor of metalloprotease activity, added extracellularly to AM + WTp0 functionally “converted” WTp0 into a “ΔLasB-like” PAO1, rendering it more sensitive to AM killing (histogram 3); second, and conversely, there was a trend, upon extra-cellular addition of LasB protein, toward the functional conversion of ΔLasBp0 into a “WT-like” PAO1 (histogram 6). Similar effects of PA and LasB protein were observed on primary lung AMs infected with either WTp0- or ΔLasBp0 (Figure S3A in Supplementary Material), independently of cytotoxicity (Figure S3B in Supplementary Material).

When we analyzed separately MPI supernatants and lysates (instead of pooling them as above), we showed that, in both compartments, there were fewer bacteria recovered following ΔLasBp0 infection (Figure 4C), and that quantitatively, the highest number of bacteria was recovered in the supernatants of cells infected with WTpLasB (Figure 4C and not shown), demonstrating that LasB was acting mainly by allowing PAO1 to survive in the supernatant.

By contrast, ΔLasBp0-PAO1 survived less in AM supernatants, and as a consequence, there was also after phagocytosis proportionally less ΔLasBp0 than WT-PAO1 bacteria in cell lysates. Again, there was no consistent correlation between the presence or absence of LasB and cell cytotoxicity (LDH activity, Figure 4D; Figures S2D and S3B in Supplementary Material), and there was no difference in AM phagocytosis (not shown).

In addition, we showed that PAO1 infection of MPI cells, regardless of the strain, significantly induced the production (mRNA assessment, Figures 4E,F) and release (protein measurement, Figures 4G,H) of IL-1β and TNF-α. IL-1β mRNA production did not correlate strictly with either the presence or absence of LasB (no statistical significance, see legend of Figure 4E). Notably, there were statistical significant differences in TNF-α mRNA production between strains, but this did not seem to be related either to the presence or not of LasB (see legend of Figure 4F). However, interestingly, whereas TNF-α protein accumulation was again independent of the presence of LasB (see legend of Figure 4H), IL-1β secretion/accumulation was clearly higher in LasB-producing strains (WTp0, WTpLasB, ΔLasBpLasB) (Figure 4G), suggesting that LasB may be activating the inflammasome in MPI cells.

### Las B Does Not Target TLR-5 Nor Flagellin-Mediated TLR-5 Signaling

Given our previous demonstration of the importance of the TLR-5-IL-1β link in AM recognition and killing of *P.a* (18), we wondered whether LasB might be able to modulate macrophage bacterial killing, either through the disruption of flagellin (FliC) activity (the only *P.a* ligand of TLR-5), as shown by Casilag et al. for AprA (22), or through direct TLR-5 impairment (e.g., through proteolysis of the receptor). We first demonstrated by q-PCR that AMs, MPI cells, and THP-1 cells express TLR-5 mRNA, albeit to varying degrees (Ct = 24, 31, 27, respectively). In accordance, MPI cells were responsive to FliC, when both TNF and IL-1β were used as read-outs (Figure S4A in Supplementary Material), confirming the presence of TLR-5 on these cells. To test whether LasB could affect TLR-5 signaling (e.g., through proteolysis), MPI cells were pretreated with either WTp0- or ΔLasB p0-PAO1 secretomes (SECs), washed, and then stimulated with the TLR-5 ligand FliC. As a result, no differential TLR-5 signaling was observed, when IL-1β and TNF were used as read-outs (Figure S4B in Supplementary Material) and Western Blot analysis indicated that there was no differential cleavage of TLR-5 by either WTp0- or ΔLasB p0-PAO1 SECs (data not shown). Altogether, this suggests that LasB (present in WTp0- but not in ΔLasB p0-PAO1 SEC) does not affect the TLR-5 receptor, nor its signaling.

**Figure 5:**
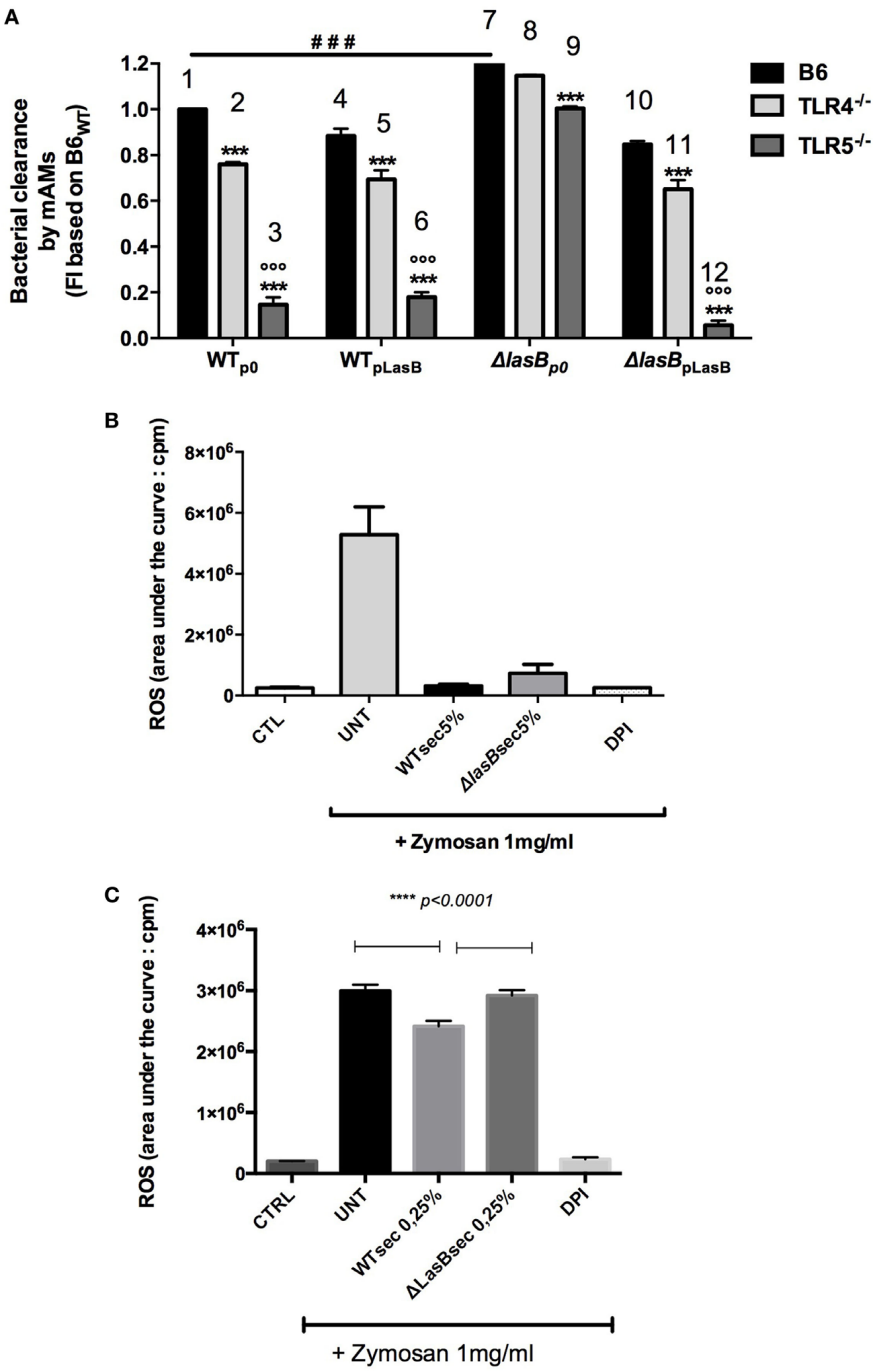
Role of TLR-5 in bacterial clearance and modulation of reactive oxygen species (ROS) production by PAO1 secretomes. **(A)** Pooled primary lung alveolar macrophages (AMs) from WT C57Bl/6JRj (*n* = 6), TLR-4 KO (*n* = 6), TLR-5 KO (*n* = 6) mice were obtained from BALs, as above. Cells (10^5^ cell in 96 wells) were infected with WTp0-, WTpLasB-, ΔLasBp0-, ΔLasBpLasB-PAO1 (moi 1) for 4 h. Then, bacterial clearance was measured by pooling supernatants and cell lysates (see Figure 4). Results are expressed as mean ± SEM and statistical analysis performed with one-way ANOVA followed by Tukey’s test. ^###^*p* < 0.001, compared to p0-PAO1 infection of C57/Bl6 AMs; ***p < 0.001, compared to B6; ^ooo^*p* < 0.001, compared to ΔLasBp0 TLR5^−/−^. **(B)** MPI cells (7 × 10^5^ cells in 5 mL tubes) were pre-incubated (*n* = 4) with either medium (CTL), WTp0- or ΔLasBp0-PAO1 SECs (5%), or with diphenyleneiodonium (an inhibitor of the NADPH oxidase). After 30 min, either medium (CTRL) or opsonized zymosan (1 mg/mL) and luminol were added to the culture medium. ROS production was recorded using chemiluminescence measurements as explained in Section “Materials and Methods.” Results are expressed as mean ± SEM and statistical analysis performed with one-way ANOVA followed by Tukey’s test. CTRL v Untreated, v WT SEC 5%, v ΔLasB SEC 5%: all *p* < 0.05; WT SEC 5%, v ΔLasB SEC 5%: *p* = 0.1. **(C)** Independently (*n* = 3) from **(B)**, a similar experiment was performed, but WT and ΔLasB SECs were used at a concentration of 0.25%, instead of 5%. Results are expressed as mean ± SEM and statistical analysis performed with one-way ANOVA followed by Tukey’s test. CTRL v Untreated, CTRL v WT SEC 0.25%, CTRL v ΔLasB SEC 0.25%: all *p* < 0.0001; WT SEC 0.25% v ΔLasB SEC 0.25%: *p* < 0.0001.

In parallel, experiments were performed *ex vivo* with WT and TLR-5 KO AMs (Figure 5). Similarly, although TLR-5 KO AMs were drastically impaired in their ability to kill WTp0- and ΔLasB-pLasB-PAO1 (when compared to WT and TLR-4 KO mice), confirming our previous study (18), the killing of ΔLasBp0- by TLR5 KO AMs was still more efficient than that of “WT-PAO1 strains” (compare histogram 9 with histograms 3, 6, 12), demonstrating that TLR-5 is not the target of LasB (Figure 5A). We also showed, as an aside, that TLR-4 is not its main target either (ΔLasBp0-PAO1 is still more killed than “WT-PAO1 strains,” compare histograms 8 with histograms 2, 5, 11). Altogether, as in the above section, these results suggested that WT-PAO1 strains likely did not interfere with AM TLR-5-mediated killing through LasB alone, and that LasB (alone or in conjunction with other components) may be acting through different mechanisms to impair AM killing of *P.a*.

### PAO1 SEC Affects Zymosan-Induced ROS Production by AMs

Based on the results above, we then checked whether LasB could target other potential pathways and or sensing receptors implicated in the recognition of flagellated bacteria, such as the complement system, IgG receptors, integrins, TLR-2, TLR-6, asialoGM1, dectin-1 (23–30).

Indeed, we showed that serum-opsonized zymosan, an activator of TLR-2, dectin-1, and of the complement cascade, induced ROS production in MPI cells (histogram “UNT”), and that expectedly DPI, a known inhibitor of the NADPH-oxidase/NOX2, very significantly blunted the production of ROS (Figure 5B). Significantly, both PAO1 strains SECs (at a concentration of 5%) drastically inhibited ROS generation induced by zymosan, and there was a trend, although not statistically significant (*p* = 0.1), for a higher downregulation of ROS production with WT than with ΔLasB-PAO1 SEC (Figure 5B). When a lower concentration of SEC was used (0.25%), WT-SECs had also a downregulatory effect on zymosan-induced ROS production, albeit of a lesser intensity, demonstrating a dose–response effect. Importantly, at that concentration of SEC, there was a statistically significant difference between WT- SEC and ΔLasB-PAO1-SECs, confirming that LasB was, at least partly, responsible for this phenomenon.

### Proteomics Analysis of MPI–PAO1 Interaction

**Figure 6:**
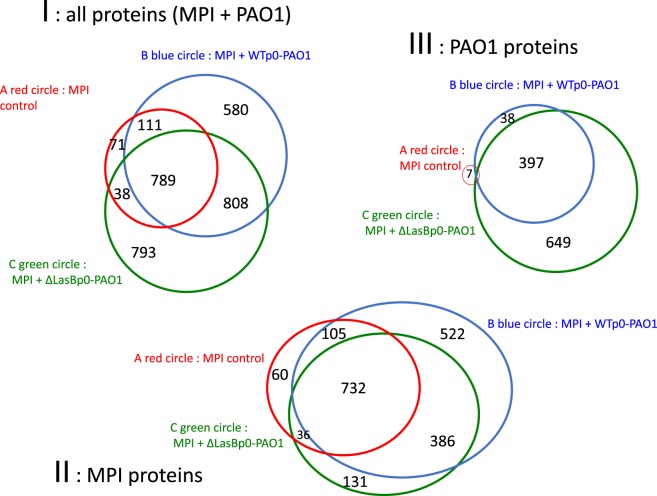
Venn Diagrams of total hits recovered in the supernatant of MPI cells, either non-infected (Control) or infected with WTp0- or ΔLasBp0-PAO1. MPI cells were either left un-infected (MPI Control, *n* = 2) or infected (moi 1) with WTp0- (*n* = 3) or ΔLasBp0-PAO1 (*n* = 3) in serum-free medium. After 4 h, supernatants were recovered, concentrated and analyzed by mass spectrometry (see Materials and Methods in Supplementary Material). For the identification step, all MS and MS/MS data were processed with the Proteome Discoverer software (Thermo Scientific, version 2.1) coupled to the Mascot search engine (Matrix Science, version 2.5.1). The NCBInr database (2016) with the *Mus musculus* and the *Pseudomonas aeruginosa* taxonomies were used for the MS/MS identification step. Peptide identifications were validated using a 1% False Discovery Rate threshold calculated with the Percolator algorithm. Protein abundance variations were measured according to the Hi-3 label-free quantification method and validated if their calculated ANOVA *p*-values were under 0.05 (see Materials and Methods in Supplementary Material). Venn diagrams generated from “Proteome Discoverer” are shown with numbers referring to the numbers of hits identified. Panel I: all proteins (MPI + PAO1); Panel II: MPI proteins only; Panel III: PAO1 proteins only. Of note, the reported seven bacterial proteins in the “MPI Control treatment” are clearly artifacts (panel III, circle A).

To further investigate the potential mechanisms of action of LasB, we chose to unbiasely analyze, at the proteomic level (using mass spectrometry, see Section “Materials and Methods”), the SECs from MPI cells infected with either WTp0- or ΔLasBp0-PAO1. The total number of supernatant proteins (MPI- and bacterial-derived) recovered was 3,190 (Figure 6, panel I). Of these, 62 and 38% were of MPI eukaryotic and bacterial origins (panels II and III, respectively). Notably, there were more MPI-derived proteins following WTp0 or ΔLasBp0-PAO1 treatment (panel II, circles B and C), compared to un-infected cells (panel II, circle A), in agreement with a cellular “response to infection.” More specifically, more MPI-derived proteins were recovered following WT-p0 infection than with ΔLasBp0-PAO1 (panel II, circle B versus circle C), demonstrating a significantly “higher response” in the former.

More bacterial proteins were recovered following MPI infection with ΔLasBp0- than with WT-strains (panel III, circle C versus circle B), suggesting that the former, which is less resistant to killing (see Figures above), may have released these proteins upon being killed.

**Figure 7:**
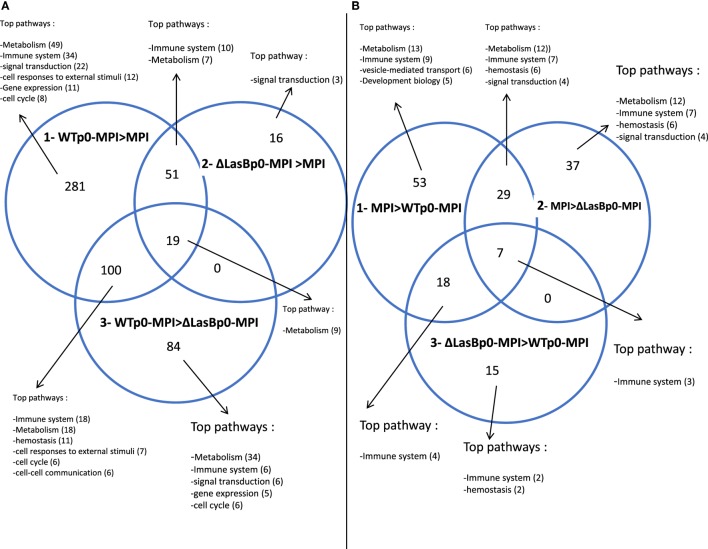
Venn diagrams of MPI hits (see Figure 6) whose expression differ by a factor of at least 2 (*p* < 0.05) between the represented conditions (see full list in Figures S5–S10 in Supplementary Material). **(A)** Circle 1: WTp0-PAO1-MPI > MPI control; circle 2: ΔLasBp0-PAO1-MPI > MPI control; circle 3: WTp0-PAO1-MPI > ΔLasBp0-PAO1-MPI. **(B)** Circle 1: MPI control > WTp0-PAO1-MPI; circle 2: MPI control > ΔLasBp0-PAO1-MPI; circle 3: ΔLasBp0-PAO1-MPI > WTp0-PAO1-MPI. For each area of the Venn Diagram circles (generated with http://bioinfogp.cnb.csic.es/tools/venny/), the top pathways identified with the Reactome program (http://reactome.org/) are indicated, with the number of hits in parenthesis.

**Figure 8:**
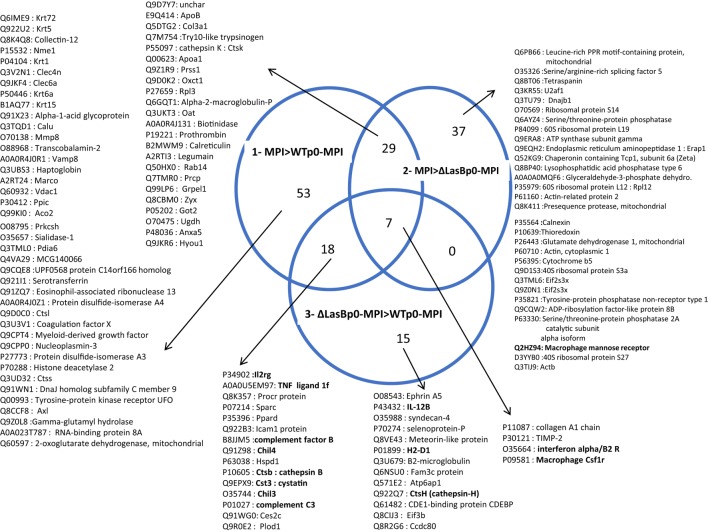
List of MPI proteins extracted from the Venn Diagrams represented in Figure 7B (see full list in Figures S5–S10 in Supplementary Material). For each area of the Venn Diagram circles, the proteins identified within the hits are represented, with their Uniprot accession number. NB: the difference between the number of hits and the number of proteins represent entities with more than one Uniprot identifier; For example, P34902 and Q3UPA9 are two different “hits” representing the same protein (IL2rg, at the intersection of circles 1 and 3), but only P34902 is listed.

The MPI-derived proteins from the different treatments (Figure 6, panel II) were then analyzed in detail [WTp0- + MPI v MPI alone; ΔLasBp0- + MPI v MPI alone; and WTp0- + MPI v ΔLasBp0-PAO1 + MPI (Figures 7 and 8; Figures S5–S10 in Supplementary Material)]. When compared side by side, the condition WTp0-PAO1 > ΔLasBp0-PAO1 (fold-increase > 2) identified higher levels of 203 proteins (100 + 19 + 84, Figure 7A, circle 3), whereas that of ΔLasBp0-PAO1 > WTp0-PAO1 (fold-increase > 2) identified higher levels of only 40 proteins (18 + 7 + 15, Figure 7B, circle 3). For the former comparison, the top pathways identified with the Reactome website were “Metabolism” (61 hits = 18 + 34 + 9), “Immune system” (24 hits = 18 + 6), “Hemostasis” (11 hits), “responses to external stimuli” (7 hits), “signal transduction” (6 hits). Notably, two nodes consisted mainly of proteins involved in transcription and translation (e.g., RpI3, 5, 35, 38, Eif3c, Eef1g, Ddx39b), or cell signaling (kinases, transcription factors such as Irak4, Syk, Stat3, Mapk1, Mapk3, Akt2, see Figure S5 in Supplementary Material). By contrast, for the latter comparison (i.e., proteins more induced by ΔLasBp0- than by WTp0-PAO1, Figure 7B, circle 3), the hierarchy in regulated pathways was different: “Immune system” (9 hits = 4 + 3 + 2), and Hemostasis (2 hits). Proteins involved in innate immune responses were particularly present here [Il-2rg, H2-D1, IL12b, Ifnar2, complement factor B, complement C3, Sepp1, ChilI3, ChilI4, macrophage Csf1r, cathepsin-B, cathepsin-H, cystatin (Figure 8, circle 3, bold; Figure S6 in Supplementary Material)]. Interestingly, most of these proteins were also found upregulated in the MPI control > WT-PAO1 + MPI condition (Figure 8, circle 1). Altogether, these data demonstrate that LasB downregulates or inactivates secreted innate immune components, including the C3 and factor B complement molecules.

### PAO1-WT Infection Decreases C3 and Factor B Protein Accumulation in MPI Supernatants

Because the proteomics analysis (see above) revealed that complement factors C3 and B levels were lower in the supernatants from AMs infected with WT-PAO1, when compared with ΔLasB-PAO1 infection and with Control un-infected treatment (suggesting a proteolytic effect of LasB on these components), their protein levels were then directly assessed by ELISA.

**Figure 9:**
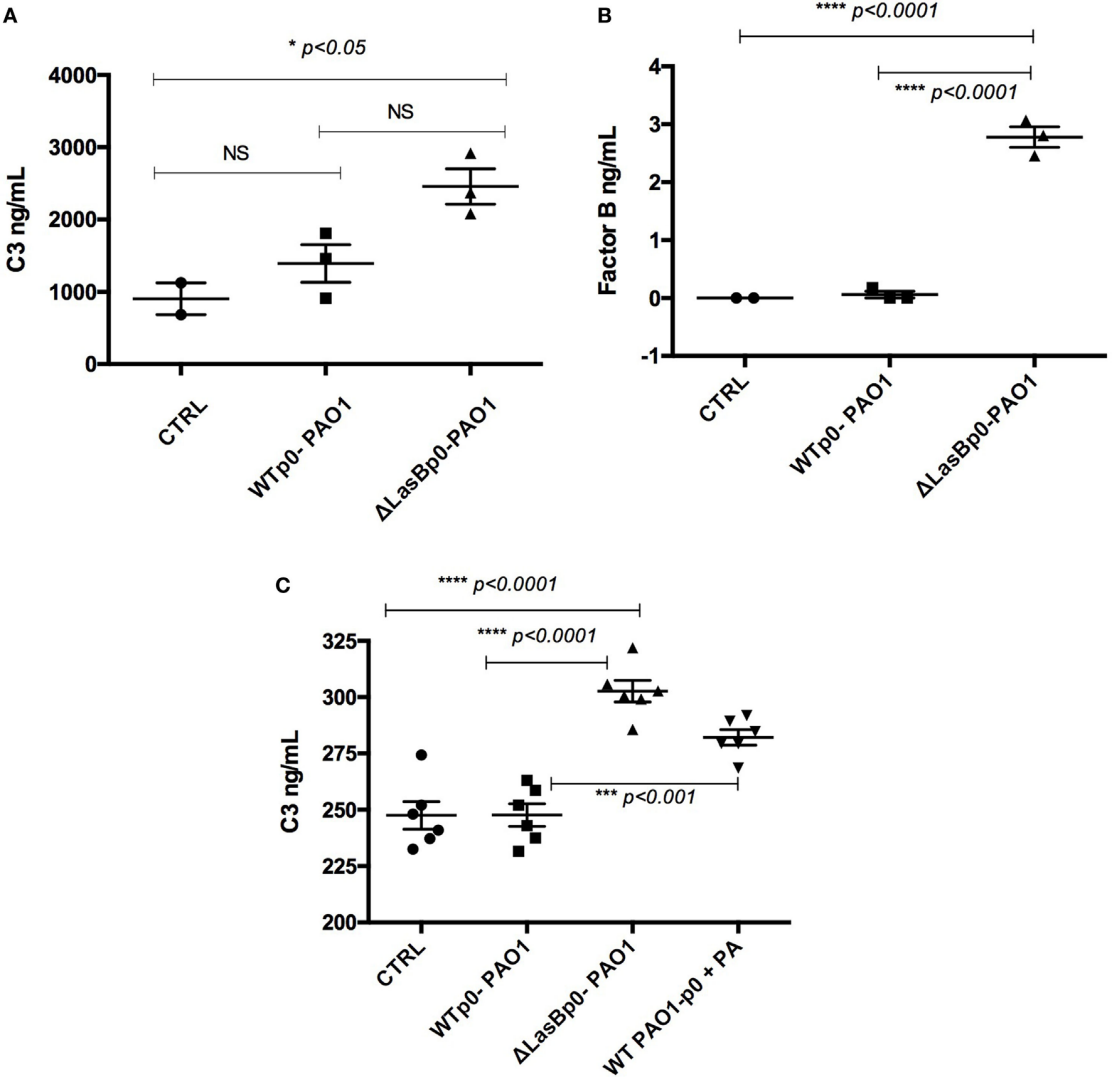
Measurement of C3 and factor B levels in MPI supernatants following WT- or ΔLasBp0-PAO1 infection. **(A,B)** “Proteomics experiment”: the same supernatants used for the Proteomics analysis (see Figure 6) were analyzed by enzyme-linked immunosorbent assay (ELISA) for C3 **(A)** and factor B **(B)** content. Results are expressed as mean ± SEM, and analyzed with one-way ANOVA, followed by Tukey’s post-analysis. **(C)** Independently of **(A,B)**, MPI cells (*n* = 3) were either left uninfected (CTRL) or infected (see legend of Figure 4) during 4 h with either WT- + p0- or ΔLasB- + p0-PAO1 (moi = 1), in the presence or not of phosphoramidon (PA) (10 µM). Supernatants were then harvested and analyzed by ELISA for C3 content. Results are expressed as mean ± SEM, and analyzed with one-way ANOVA, followed by Tukey’s post-analysis.

In the same supernatants used for the Proteomics analysis, we showed that Control AMs produced considerable amounts of C3 (1,000 ng/mL levels), and that ΔLasB-PAO1 infection upregulated that production, presumably through PAMPs stimulation of AM receptors (Figure 9A). By contrast, there was a trend toward reduced amounts of C3 upon infection with WT-PAO1, strongly suggesting that the presence of LasB in WT-PAO1 was responsible for degrading the PAMPs-induced secretion of C3.

When complement factor B was considered, Control AMs did not produce detectable amounts of the molecule, but as for C3, ΔLasB-PAO1 infection upregulated that factor, albeit to lesser levels (from 0 to 3 ng/mL, Figure 9B).

We confirmed, in an independent set of experiments (*n* = 3), that indeed ΔLasB-PAO1 infection induced higher levels of C3 and that the downregulation with WT-PAO1 was proteolytically mediated since PA, the LasB inhibitor, partly reversed the phenotype. Indeed, in that condition, roughly the same amount of C3 was recovered following either WT-PAO1 + PA or ΔLasB-PAO1 infection of MPI cells (Figure 9C).

Notably, the levels of C3 recovered in the “Proteomics experiment” (panel A) were roughly 10 times higher than with the experiments depicted in panel C. This can be explained by the fact that more cells were used in the former experiment, and that the derived supernatants were concentrated for the “Proteomics protocol” (see Materials and Methods). In keeping with this, we could not detect factor B in the independent set of experiments (*n* = 3), presumably because of the higher threshold level of detection in the factor B ELISA (1 ng/mL).

### Purified *P.a* LasB Treatment of AMs Affects Clearance of *S. pneumoniae*

Given that we showed above that LasB could downregulate complement activity, we then assessed whether LasB pretreatment of MPI could affect the clearance of another bacterium, *S. pneumoniae*, also known to be sensitive to complement activation.

**Figure 10:**
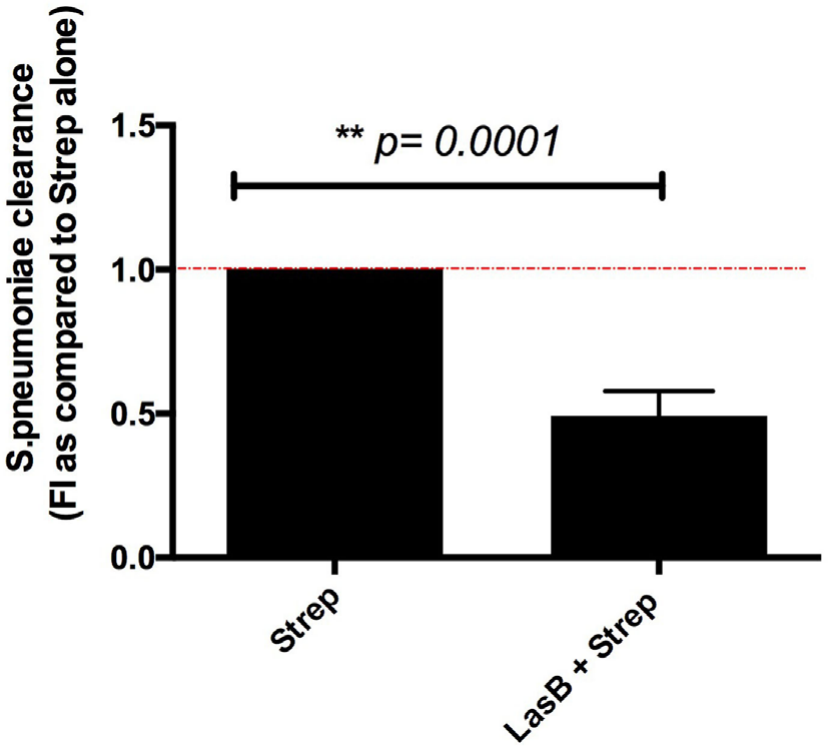
Analysis of *Streptococcus pneumoniae* clearance by MPI cells following purified LasB treatment. MPI cells were plated as described in Figure 4 (*n* = 3). They were incubated for 2 h with either medium alone (serum-free RPMI) or medium supplemented with 50 nM recombinant LasB. After washing, cells were infected in serum-free RPMI with *S. pneumoniae* (MOI 1) for 4 h. In parallel, the bacterial inoculum was seeded at T0 in the absence of macrophages as a control of bacterial growth. For bacterial count and bacterial clearance measurements, supernatants were collected and cells were lysed in PBS-Triton as explained in Section “Materials and Methods.” 100 µL of each treatment (at various dilutions) were spread on THYB-Agar plates and left 48 h at 37°C, 5% CO_2_ for CFU count. As in Figure 4, a value of 1 was given to the maximal bacterial clearance obtained with the *S. pneumoniae* alone, and the LasB + *S. p* treatment was then expressed relative to 1. Results are expressed as mean ± SEM, and statistical significance analyzed with *t*-test.

Indeed, we showed (Figure 10) that a pretreatment of MPI with LasB significantly reduced the clearance of that bacterium, 4 h postinfection, suggesting that *P.a* LasB might contribute to increased co-bacterial infections in the lung.

## Discussion

We show here in mice survival experiments that LasB-expressing PAO1 strains were more virulent than ΔLasB-PAO1 (Figure 1), and that mice infected intra-nasally with WT-PAO1 had a higher bacterial load as early as 2 h postinfection, a time point when the “inflammatory wave” is still nascent, and where epithelial cells and AMs should have a prevalent role (see Figures 2 and 3). Indeed, Cowell et al. have shown that a LasB-*P.a* secreting strain was associated with increased invasivity in rabbit corneal epithelial cells (31), and we and others have shown that T2SS (and in some cases specifically LasB) could downregulate *in vitro* a variety of epithelial-derived protective molecules including surfactant (17, 32, 33), cytokines, antimicrobial molecules, and receptors (5, 14, 34–48). Although our *in vivo* results can be partly explained by an effect of LasB on lung epithelial cells (5), we also considered here the role of the alveolar macrophage, a cell type which had seldomly been considered previously in that context (49–51), and never, to our knowledge, in relation specifically to responses to LasB. Indeed, we observed, using macrophage cell lines as well as primary BMDMs and AMs, that these cells killed fewer WT than ΔLasB-PAO1 bacteria, demonstrating that LasB had a clear subverting effect on AM function. Although we cannot completely rule out a potential additional intra-cellular activity, we believe that most of its activity is probably extra-or peri-cellular-mediated based on the fact that many more bacteria are recovered in the supernatant of macrophages infected with WT-PAO1 bacteria (Figure 4C and not shown), suggesting an effect of LasB prior to phagocytosis. Because of the importance of AM TLR-5 in *P.a* killing (18), we tested whether LasB may be acting on that important receptor: we confirmed that AM TLR-5 is indeed essential for eliminating bacteria, but showed the phenomenon to be LasB-independent (Figure 5A; Figure S4 in Supplementary Material). Relatedly, Bardoel et al. showed in *in vitro* experiments that AprA (an alkaline protease also secreted through the T2SS of *P.a*) could degrade flagellin and impair epithelial cell (HEK-293 cells) TLR-5 signaling (52). However, a direct degrading effect of LasB on flagellin in our studies is unlikely since: (1) it would have the same “functional” net effect as using TLR-5 KO cells (see above); (2) WT-PAO1 strains SECs (known to contain FliC monomers) are more potent than ΔLasB SECs (Figure 4G) at inducing the TLR-5-dependent cytokine IL-1β (18), therefore, probably ruling out a LasB-dependent degradation of FliC in our system.

In addition to TLR-5, some studies have shown in other systems (albeit not in AMs) that TLR-2 and dectin, among other receptors, may also be implicated in the recognition of flagellated bacteria such as *P.a* (23–30). We showed here that the likely activation of (at least) dectin, TLR-2, and of the complement system with serum-opsonized zymosan was drastically blunted by both PAO1 SECS (as indicated by reduced ROS production), and that LasB contributed to the effect (Figures 5B,C). Because serum-opsonized zymosan is known to activate the alternative pathway of the complement cascade (through the generation of the C3 convertase C3bBb), an additional action of LasB on complement components was also possible in our system. In accordance, our Proteomics analysis revealed that among the top innate immune components hits modulated by LasB (including ChiL3/YM-1, ChiL4/YM-2, cathepsin B, cathepsin H, IL12b, Ifnar2, Csf1r, etc.), C3 and factor B were found to be more downregulated by WT-PAO1 SEC than by ΔLasB SEC (Figure 8, circle 3, Figure 9; Figure S6 in Supplementary Material), altogether suggesting that these mediators are likely important innate immune targets for WT-PAO1 subversive strategies.

This subversive effect of LasB on the complement activity, demonstrated in AMs, echoes earlier data obtained *in vitro* with purified proteins, which showed that C1q and C3 were targets of *P.a* AprA, and (to a lesser extent) of Las B (37). Furthermore, in a different system, AprA was shown to inhibit neutrophil opsonization of *P.a, via* cleavage of the complement factor C2 (53), confirming that *P.a* can target the complement system to manipulate host responses. Our data, which show that *P.a* has developed strategies to circumvent the activity of C3 and B, key factors involved in both the classical and alternative complement pathways, confirm the importance of this pathway in the fight against enveloped Gram-bacteria, including *P.a* (54–56). Our proteomics data also confirm that monocytes/macrophages are an important source of active C3 and factor B, as originally described in the 1980s (57, 58). Because the complement system is also important in the fight against *S. pneumoniae*, another lung pathogen (59), we tested and indeed demonstrated that LasB significantly impaired the clearance of *S. pneumoniae* by MPI cells (Figure 10). This may have implications for the role of LasB in favoring lung co-infections with *S. pneumoniae* and *P.a* since both bacteria have indeed been reported in pneumonia (60–62).

In conclusion, our study is to our knowledge, the first to report a direct subversive effect of LasB on unopsonized alveolar macrophage activity, leading to the worsening of *P.a* infection *in vitro* and *in vivo*, and contributing to the death of experimental animals. Although the molecular mechanisms underlying the effect of LasB on macrophages are clearly multiple, we have demonstrated that neither TLR-5, an important receptor for AM killing of *P.a* [(18); Figure 5; Figure S11 in Supplementary Material], nor TLR-4, are targets of LasB. Instead, as demonstrated in our Proteomics analysis, the latter targets instead secreted innate immune molecules such as complement factors (C3, factor B), cytokines (TNF ligand 1f, IL-12, etc.), repair molecules (Chil-3/YM-1, Chil-4/YM-2), and alternative immune receptors (IL2Rγ, Csf1r, ICAM-1, IFNAR).

These targets are altogether referred as “R” in the summarizing Figure S11 in Supplementary Material and their downregulation probably concur to downregulate *P.a* clearance and increase bacterial virulence. Our data further suggest that the AM should also be considered as an important therapeutic target in conditions such as nosocomial pneumonia or cystic fibrosis, where *P.a* is prevalent (2, 63, 64).

## Ethics Statement

Procedures involving mice were approved by our local ethical committee (Paris-Nord/No 121) and by the French Ministry of Education and Research (agreement number 04537.03).

## Author Contributions

J-MS conceived the projet, analyzed data, and wrote the manuscript. FB, SK, and BV performed the experiments and analyzed data. VS-C, PD, JE-B, J-CS, and RV provided crucial help as well as key reagents, bacterial strains, and murine lines.

## Conflict of Interest Statement

The authors declare that the research was conducted in the absence of any commercial or financial relationships that could be construed as a potential conflict of interest.

## Footnotes

^1^http://bioinfogp.cnb.csic.es/tools/venny.

^2^https://string-db.org/.

^3^https://reactome.org/.
