# Supplementary material for: Pseudomonas aeruginosa LasB Subverts Alveolar Macrophage Activity by Interfering With Bacterial Killing Through Downregulation of Innate Immune Defense, Reactive Oxygen Species Generation, and Complement Activation
Source: Front Immunol. 2018 Jul 23;9:1675. doi: 10.3389/fimmu.2018.01675 (PMC6064941; doi:10.3389/fimmu.2018.01675)
Supplement: Supplementary file 1 [file Data_Sheet_1.zip › 07-12-2018_10.3389-fimmu.2018.01675/Figure_S1.PPTX]

## Slide 1
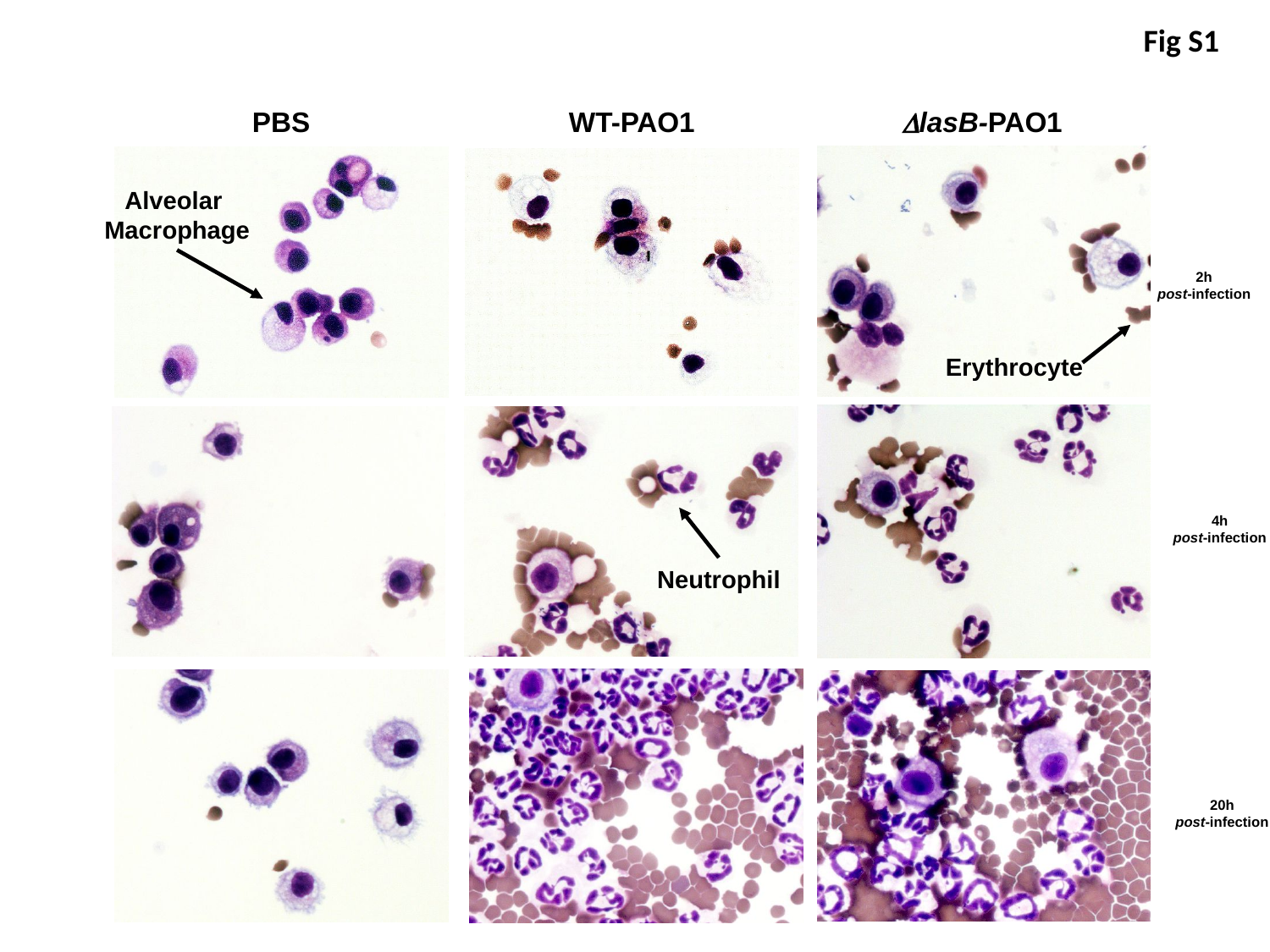

Fig S1
PBS
WT-PAO1
DlasB-PAO1
2h
post-infection
4h
post-infection
20h
post-infection
Alveolar
Macrophage
Erythrocyte
Neutrophil
