# Supplementary material for: Pseudomonas aeruginosa LasB Subverts Alveolar Macrophage Activity by Interfering With Bacterial Killing Through Downregulation of Innate Immune Defense, Reactive Oxygen Species Generation, and Complement Activation
Source: Front Immunol. 2018 Jul 23;9:1675. doi: 10.3389/fimmu.2018.01675 (PMC6064941; doi:10.3389/fimmu.2018.01675)
Supplement: Supplementary file 1 [file Data_Sheet_1.zip › 07-12-2018_10.3389-fimmu.2018.01675/Supplementary Figure Legends.docx]

**SUPPLEMENTARY FIG LEGENDS:**

**Fig S1: BAL cell differential (cytospins obtained at 2, 4, and 24hrs) from mice instilled with either PBS, WT-PAO1 (5.10^6^ pfu) or ΔLasB-PAO1 (5.10^6^ pfu)**

BAL fluids obtained from mice instilled with either PBS, WT-PAO1 (5.10^6^ pfu) or ΔLasB-PAO1 (5.10^6^ pfu) (see Fig 2) were cytospined and stained with Diff-Quick. Alveolar macrophages, neutrophils and erythrocytes are indicated with arrows (magnification X100).

**Fig S2: Analysis of bacterial clearance by mouse primary alveolar macrophages (AMs) and macrophage cell lines**

A) In a ‘typical experiment’, 10^5^  MPI cells (in 96 well plates) were infected in serum-free RPMI medium during 4hrs (moi = 1) with either WT-PAO1-p0 (WT-p0 = Ø), WT-p0 + pLasB, WT-pLasB +PA (phosphoramidon/ PA 8.5 μM) or with ΔLasB+p0; ΔLasB+pLasB, ΔLasB-p0+ purified LasB (10nM). Then, supernatants and cell lysates (S + L) were pooled and CFU of remaining bacteria were counted on LB-agar plates. Results are expressed as means+/- SEM (technical triplicates). These experiments, repeated 5 times, are presented in a different format (‘Pseudomonas clearance’ instead of absolute bacterial counts) in Fig 4A.

B) Primary mouse alveolar macrophages (mAMs, obtained from naïve lavaged C57/Bl6 mice), MHS, MPI, and THP-1 cells (see Materials and Methods) were plated (10^5^ cells in 96 well plates) and infected with either WTp0-, ΔLasBp0-PAO1 (n=5, moi = 1) in serum-free RPMI medium during 4hrs. Supernatants and cell lysates (S + L) were then pooled and CFU of remaining bacteria were counted on LB-agar plates.

This was compared to the CFU obtained when the inoculum was seeded at T0 in the absence of macrophages. For bacterial clearance, a value of 1 was given to macrophages infected with WTp0-PAO1, and other treatments were then expressed relative to 1.

Statistical significance : Results are expressed as means+/- SEM, and statistical significance assessed by Mann-Whitney test.   ***: p<0.001, compared to mAMs infected by WT-PAO1, ##: p<0.01, compared to MHS infected byWT-PAO1, °°°: p<0.001, compared to MPI infected by WT-PAO1, and $$$: p<0.001, compared to THP-1 infected by WT-PAO1.

C) Primary bone marrow-derived macrophages (n=1), generated as described in Material and Methods, were plated and infected as in A). PAO1 strains clearance was assessed as in A). Error bars indicate technical triplicates (mean +/- SD).

D) Measurement of cell cytotoxicity in samples generated in A) was performed, as detailed in Materials and Methods.

**Fig S3: Analysis of bacterial clearance by mouse primary alveolar macrophages (AMs), with or without purified LasB or phosphoramidon**

A) Mouse AMs (mAMs) cells were infected (n=8, moi =1) with either WTp0-PAO1 alone (histogram 1, given a value of 1), with WTp0-PAO1 in the presence of phosphoramidon (PA 8.5μM, histogram 2), or with ΔLasBp0-PAO1 alone (histogram 3), or ΔLasBp0-PAO1 in the presence of purified LasB protein (10nM, histogram 4). Results are expressed as means+/- SEM, and statistical significance assessed by Mann-Whitney test.  **: p<0.001, between treatments 2 and 4 ; ##: p<0.01, between treatments 1 and 2.

B) Measurement of cell cytotoxicity in samples generated in A) was performed, as explained in Materials and Methods.

**Fig S4 : Flagellin-mediated TLR-5 signalling in MPI cells**

1. MPI cells (2.10^5^ cells in 96 wells) were left unstimulated or were stimulated with WT-flagellin (10ng/mL) in serum-free RPMI medium (n=3). After 4 hours, supernatants were recovered and analysed by ELISA for IL-1β or TNFα content. Results are expressed as means+/- SEM, and statistical significance compared to control was assessed by t-test  *: p<0.05.
2. MPI (2.10^5^ cells in 96 wells in serum- and antibiotic free- RPMI medium were

stimulated with either WT-PAO1-or ΔLasB-PAO1-SEC (5%) during 4 hrs in serum-free RPMI medium (n=3). After washing with PBS, cells were then stimulated with WT-flagellin (10ng/mL) in serum-free RPMI medium (n=3). After 4hrs, cells supernatants were then harvested and analysed by ELISA for IL-1β and TNFα content.

**Figs S5-S10 : Identification of MPI proteins with differential expression (fold increase >2, p<0.05) between the following conditions :** WTp0-MPI> ΔLasBp0-MPI (Fig S5) ; ΔLasBp0-MPI > WTp0-MPI (Fig S6) ; MPI control >WTp0-MPI (Fig S7) ; MPI control >ΔLasBp0-MPI (Fig S8) ; WTp0-MPI >MPI control (Fig S9) ; ΔLasBp0-MPI >MPI control (Fig S10).

**Fig S11: Implication of TLR-5 in *Pseudomonas aeruginosa* killing, and targeting of alternative receptor(s)/pathway(s) by LasB.**

TLR-5 is very important for the killing of flagellated *Pseudomonas aeruginosa* (*P.a)*  , by alveolar macrophages, as shown by Descamps et al (18), and as confirmed in the present study, with a different strain (PAO1 instead of PA-14), and using different methods. WT-*P.a* is able, however, through the secretion of LasB (red circle), to disable alternative receptor (s) (orange circle : R) or pathway(s) (secreted cytokines, C3 and factor B complement molecules) participating in *P.a* clearance. In the presence of LasB, this alternative R receptor/pathway is disabled (including reduced C3 and factor B levels), and R controls *P.a* growth less efficiently. In the presence of TLR-5, but in the absence of LasB (ΔLasB), both TLR-5 and R are active and are able to limit the growth of *P.a*. In the absence of TLR-5 and in the presence of LasB (WT-*P.a*), the disabling of R is sufficient to allow for *P.a* multiplication. In the absence of both TLR-5 and LasB, the presence of R is sufficient to control efficiently *P.a* growth.
