# Supplementary figures and images for: Pseudomonas aeruginosa LasB Subverts Alveolar Macrophage Activity by Interfering With Bacterial Killing Through Downregulation of Innate Immune Defense, Reactive Oxygen Species Generation, and Complement Activation
Source: Front Immunol. 2018 Jul 23;9:1675. doi: 10.3389/fimmu.2018.01675 (PMC6064941; doi:10.3389/fimmu.2018.01675)

## Slide 1
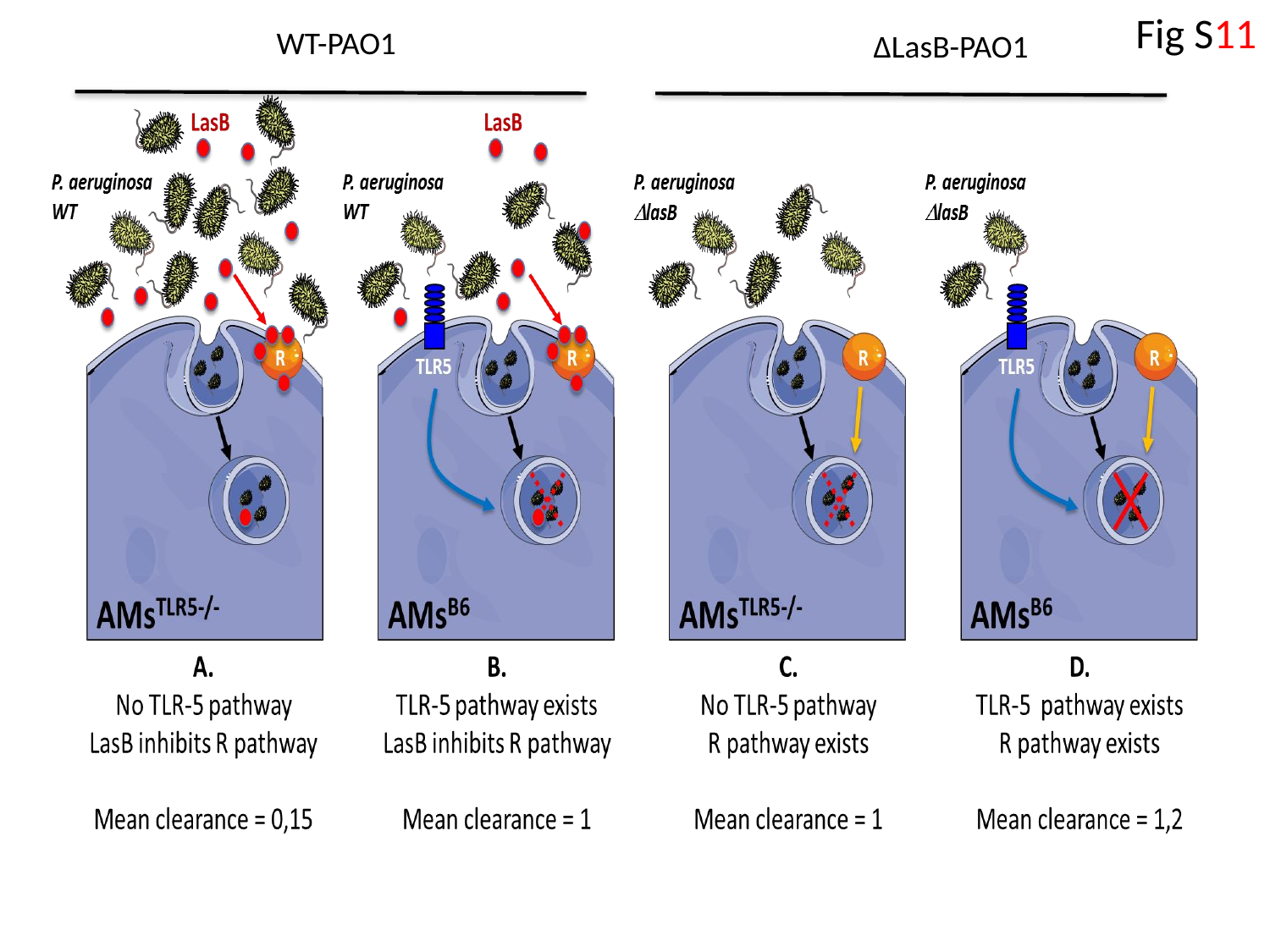

Fig S11
WT-PAO1
ΔLasB-PAO1

Supplement: Supplementary file 1 [file Data_Sheet_1.zip › 07-12-2018_10.3389-fimmu.2018.01675/Figure_S11.PPTX]

## Slide 1
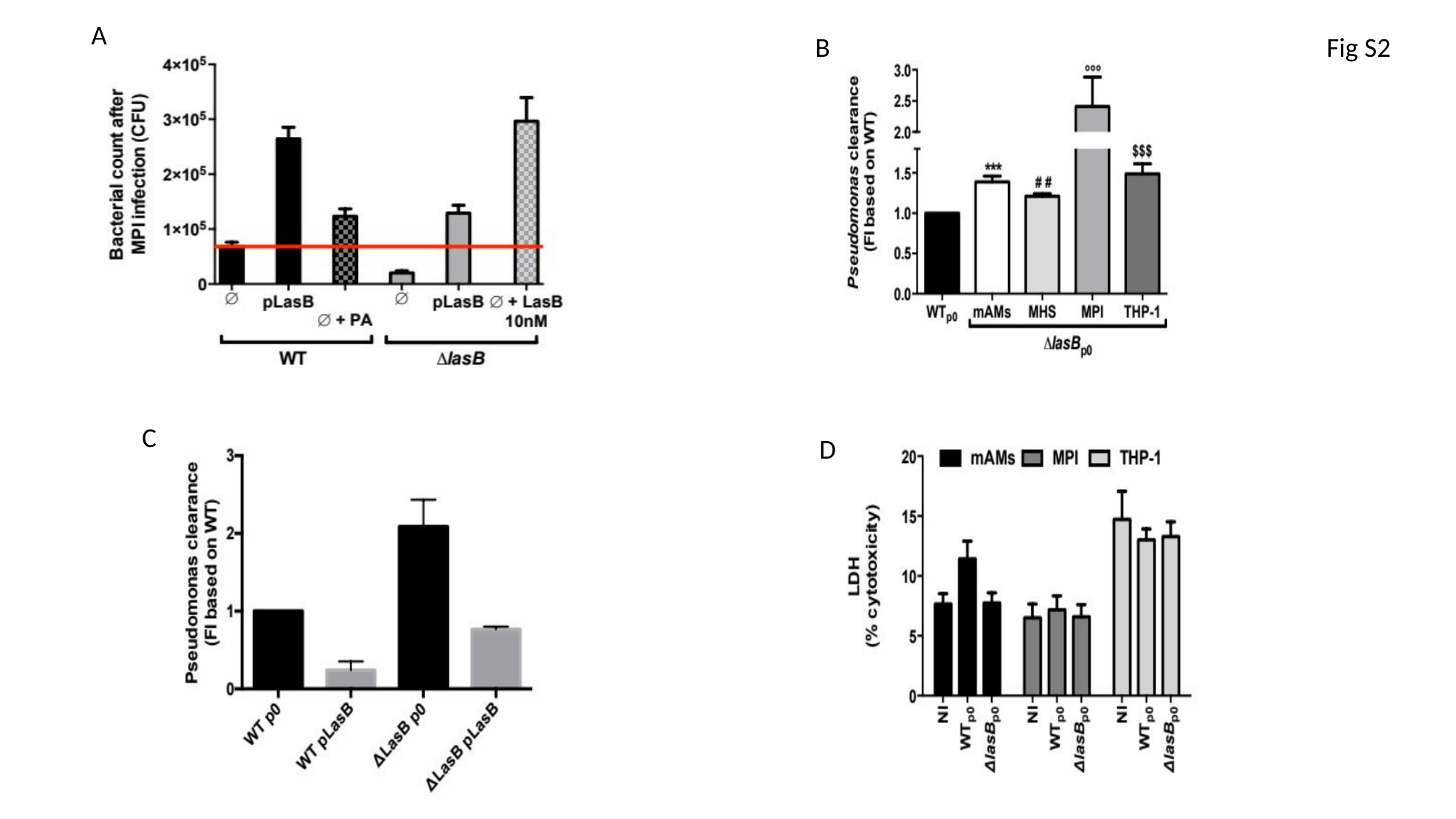

A
B
Fig S2
C
D

Supplement: Supplementary file 1 [file Data_Sheet_1.zip › 07-12-2018_10.3389-fimmu.2018.01675/Figure_S2.PPTX]

## Slide 1
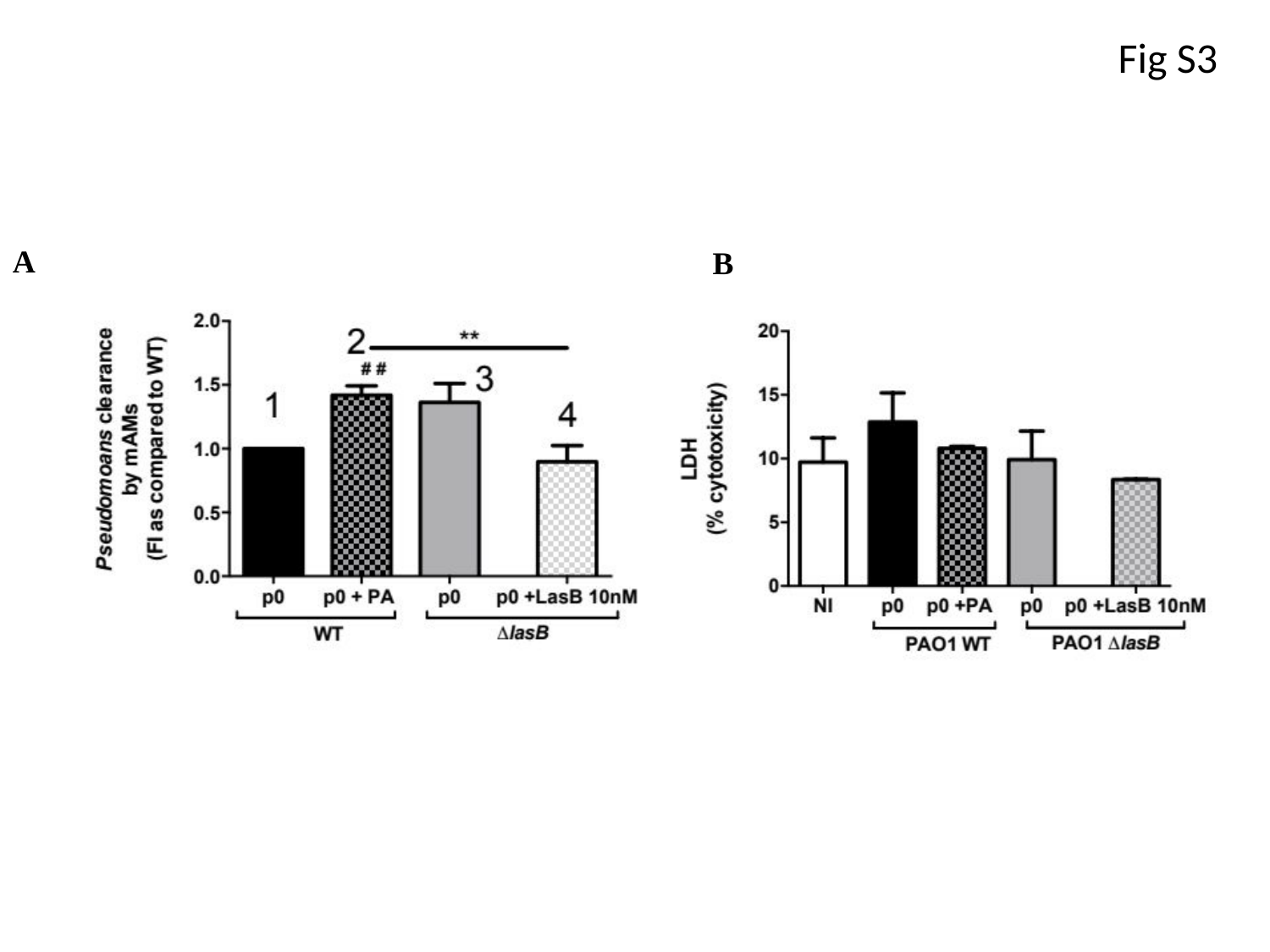

Fig S3
A
B

Supplement: Supplementary file 1 [file Data_Sheet_1.zip › 07-12-2018_10.3389-fimmu.2018.01675/Figure_S3.PPTX]

## Slide 1
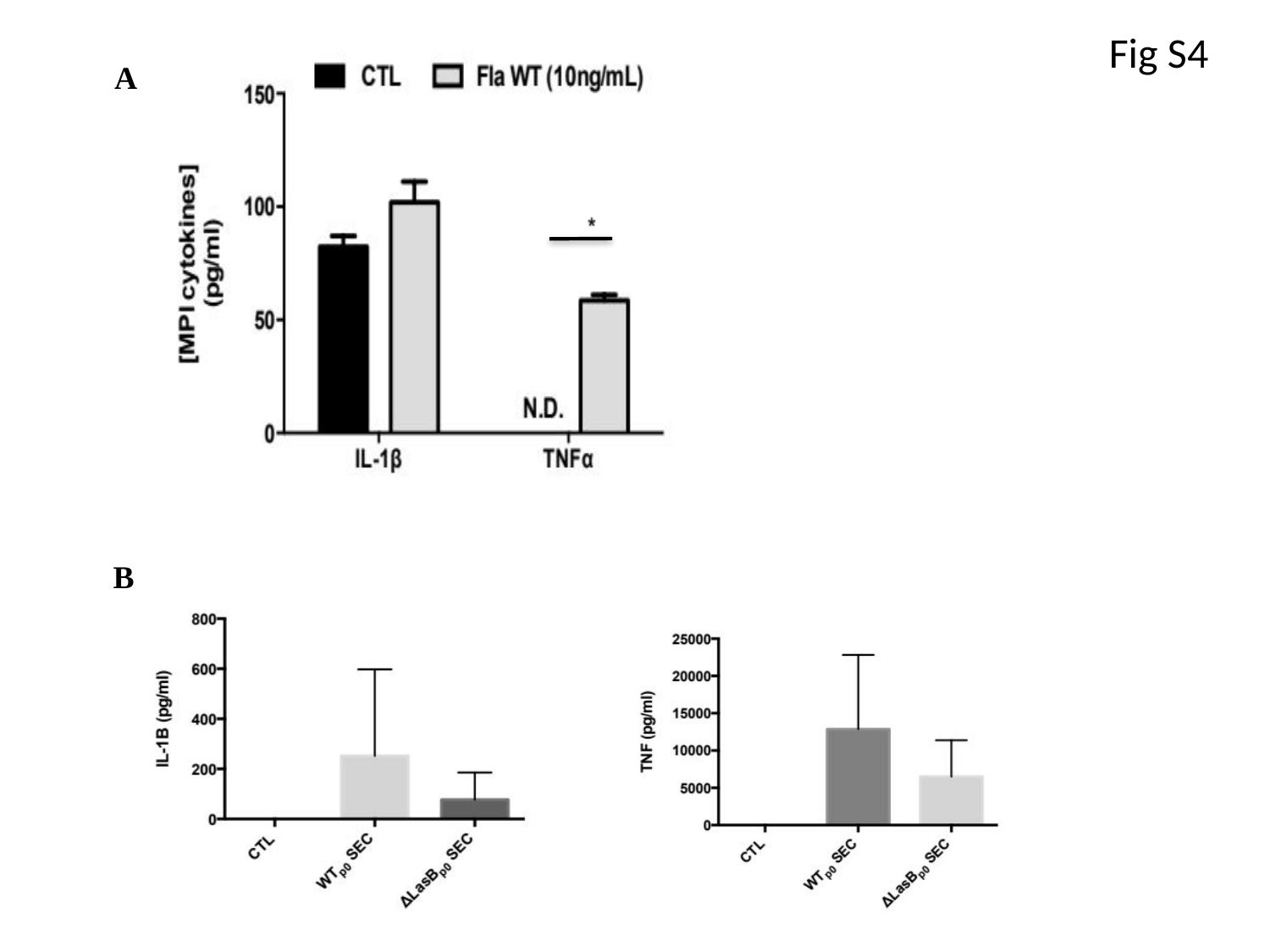

Fig S4
A
B

Supplement: Supplementary file 1 [file Data_Sheet_1.zip › 07-12-2018_10.3389-fimmu.2018.01675/Figure_S4.PPTX]
